# Detailed Evolutionary Analyses of the F Gene in the Respiratory Syncytial Virus Subgroup A

**DOI:** 10.3390/v13122525

**Published:** 2021-12-15

**Authors:** Mariko Saito, Hiroyuki Tsukagoshi, Mitsuru Sada, Soyoka Sunagawa, Tatsuya Shirai, Kaori Okayama, Toshiyuki Sugai, Takeshi Tsugawa, Yuriko Hayashi, Akihide Ryo, Makoto Takeda, Hisashi Kawashima, Nobuhiro Saruki, Hirokazu Kimura

**Affiliations:** 1 Gunma Prefectural Institute of Public Health and Environmental Sciences, Maebashi-shi 371-0052, Japan; saito-mariko@pref.gunma.lg.jp (M.S.); tsuka-hiro@pref.gunma.lg.jp (H.T.); saruki-n@pref.gunma.lg.jp (N.S.); 2 Department of Health Science, Gunma Paz University Graduate School, Takasaki-shi 370-0006, Japan; sada@paz.ac.jp (M.S.); s.sunagawa220@gmail.com (S.S.); okayama@paz.ac.jp (K.O.); hayashi@paz.ac.jp (Y.H.); 3 Department of Respiratory Medicine, Kyorin University School of Medicine, Mitaka-shi 181-8611, Japan; dw57td@bma.biglobe.ne.jp; 4 Division of Nursing Science, Hiroshima University, Hiroshima-shi 734-8551, Japan; tsugai@hiroshima-u.ac.jp; 5 Department of Pediatrics, Sapporo Medical University School of Medicine, Sapporo-shi 060-8543, Japan; tsugawat@sapmed.ac.jp; 6 Department of Microbiology, Yokohama City University School of Medicine, Yokohama-shi 236-0004, Japan; aryo@yokohama-cu.ac.jp; 7 Department of Virology, National Institute of Infectious Diseases, Musashimurayama-shi 208-0011, Japan; mtakeda@niid.go.jp; 8 Department of Pediatrics, Tokyo Medical University, Shinjuku-ku 160-0023, Japan; hisashi@tokyo-med.ac.jp

**Keywords:** respiratory syncytial virus, F gene, evolution

## Abstract

We performed evolution, phylodynamics, and reinfection-related antigenicity analyses of respiratory syncytial virus subgroup A (RSV-A) fusion (F) gene in globally collected strains (1465 strains) using authentic bioinformatics methods. The time-scaled evolutionary tree using the Bayesian Markov chain Monte Carlo method estimated that a common ancestor of the RSV-A, RSV-B, and bovine-RSV diverged at around 450 years ago, and RSV-A and RSV-B diverged around 250 years ago. Finally, the RSV-A F gene formed eight genotypes (GA1-GA7 and NA1) over the last 80 years. Phylodynamics of RSV-A F gene, including all genotype strains, increased twice in the 1990s and 2010s, while patterns of each RSV-A genotype were different. Phylogenetic distance analysis suggested that the genetic distances of the strains were relatively short (less than 0.05). No positive selection sites were estimated, while many negative selection sites were found. Moreover, the F protein 3D structure mapping and conformational epitope analysis implied that the conformational epitopes did not correspond to the neutralizing antibody binding sites of the F protein. These results suggested that the RSV-A F gene is relatively conserved, and mismatches between conformational epitopes and neutralizing antibody binding sites of the F protein are responsible for the virus reinfection.

## 1. Introduction

The respiratory syncytial virus (RSV) belongs to the genus Orthopneumovirus and the family Pneumoviridae, and causes respiratory illness in humans [1]. Particularly, the agent is responsible for severe bronchitis, bronchiolitis, and pneumonia in early infants [2,3,4]. Moreover, primary infection of the virus in infants may frequently show wheezy lower respiratory infections [5]. Epidemiological data suggest that all infants with RSV are infected by the age of 2 years [5]. Therefore, the infection caused by the virus is a major disease burden in infants and the elderly [6,7].

RSV has two structural proteins: fusion proteins (F) and attachment glycoprotein (G) on the virion surface. Among these, the F protein plays important roles in infection for the virus through the TLR4 (an inert immunity-related ligand) on the host cells and acts as a major antigen [1]. A pharmaceutical production of monoclonal antibody (palivizumab) is used as a preventive drug against RSV infection [1,8]. This drug can bind to the F protein resulting in the neutralization of the virus. Therefore, the F protein is a target molecule for vaccine development [9].

There are two F protein forms, i.e., pre- and post-fusion types [1]. Previous reports show that the prefusion type undergoes conformational changes resulting in formation of the post-fusion type [10,11]. Furthermore, a previous report indicates that the prefusion type shows a stronger antigenicity than the post-fusion type [12]. Moreover, it is suggested that neutralization of the RSV in vitro is observed when neutralizing antibodies bind to the prefusion type [11]. Although RSV reinfections occur throughout life [1], relationships between RSV antigenicity and reinfection remain unclear [13].

Phylogenetically and genetically, RSV is classified into two subgroups (RSV-A and B) and many genotypes. Previous reports suggested that RSV-A is a major prevalent subgroup, including eight genotypes [14]. Other reports also showed that some genotypes of RSV-A caused epidemics in various areas [15,16,17,18]. However, the phylodynamics of RSV-A are not exactly known.

Various in silico techniques combined with bioinformatics have been developed, and these allow us to understand detailed viral evolution [19]. In the present study, we presented evolution, phylodynamics, and reinfection-related antigenicity data of RSV-A F gene globally collected strains.

## 2. Materials and Methods

### 2.1. Strains Used in This Study

We collected full-length nucleotide sequences of the F gene of RSV-A from GenBank (https://www.ncbi.nlm.nih.gov/genbank/) to analyze the molecular evolution on 30 June 2020. In total, we obtained sequences of 4256 RSV strains. First, we selected subgroup A strains, then we omitted strains with ambiguous sequences and sequences that were not accompanied by information on the detection year and region. We also omitted 100% nucleotide sequence similarity in the same detection year and country. As a result, we used a total of 1465 strains in the analysis. All strains used in this study are shown in Supplementary Table S1.

### 2.2. Phylogenetic Analysis and Estimation of Evolutionary Rate

We used the Bayesian Markov chain Monte Carlo (MCMC) method in BEAST package v2.4.8, as previously described, to construct a phylogenic tree and estimate the evolutionary rate of the RSV-A F gene [20,21,22]. In this phylogenetic analysis, we used all the RSV-A strains (1465 strains) and two reference strains, CH18537 (a prototype RSV-B strain, accession no. JX198143) and RB94 (a prototype bovine-RSV strain, accession no. D00953). We used the jModelTest 2.1.10 programs [23] to select an appropriate substitution model, and Standard_TIM2 was selected. We performed path sampling to determine the best of four clock models (strict clock, relaxed clock exponential, relaxed clock log-normal, and random local clock) and two tree prior models (coalescent constant population and coalescent exponential population), using the Path sampler implemented in BEAST. A strict clock and the coalescent exponential population were selected. The MCMC chains consisted of 100,000,000 steps with sampling every 5000 steps. Tracer v1.7 was used to confirm the convergence of all parameters (effective sample size values above 200) [24]. After discarding the 10% burn-in, phylogenetic trees were constructed with TreeAnnotator v2.4.8 and illustrated by FigTree v1.4.0. In addition, the rates of molecular evolution were also calculated by suitable models selected for each dataset as described above.

### 2.3. Bayesian Skyline Plot Analyses

Bayesian skyline plot (BSP) analyses were performed using BEAST v2.4.8 to analyze the effective population size of the RSV-A strains and each genotype [20]. The best substitution and clock models were selected as described above. The Bayesian skyline plots were visualized with 95% highest probability density (HPD) using Tracer v1.7 [24].

### 2.4. Similarity Plot Analyses and Calculation of the Phylogenetic Distances

We calculated the nucleotide similarity of each sequence using SimPlot program 3.5.1 to clarify the relationships among the aligned nucleotide sequences of the RSV-A F gene [25]. The Long strain (a prototype RSV-A strain, accession no. JX198112) was used as the query sequence. The similarity was calculated using the Kimura 2-parameter method with a window size of 200 nucleotides and a step size of 20 nucleotides.

We constructed a phylogenetic tree of all RSV-A strains based on the maximum likelihood (ML) method using MEGA7 software to estimate the phylogenetic distance [26]. We used the jModelTest 2.1.10 programs to determine the best substitution model. Subsequently, the phylogenetic distance of the ML tree was calculated using the Patristic program [27].

### 2.5. Selective Pressure Analyses

We tested using Datamonkey (http://www.datamonkey.org/) whether sites in the F gene were under positive or negative selection as previously described on 30 June 2020 [21]. Datamonkey has an upper limit on the number of computable sequences, so it is necessary to reduce this to 500. First, the sequences with 100% amino acid sequence similarity were deleted from the dataset (552 sequences), and 52 sequences were randomly selected and deleted from the sequences with a difference of 1 amino acid to obtain 500 sequences. We used four different methods: the single likelihood ancestor (SLAC) method, the fixed effects likelihood (FEL) method, the internal fixed effects likelihood (IFEL) method, and the mixed-effects model of evolution (MEME). Significance level was *p* < 0.05.

### 2.6. Prediction of Conformational B-Cell Epitope and Amino Acid Substitution Sites by Mapping on the Structure of the RSV-A F Protein

Structural models of the prefusion F protein of RSV-A were constructed for representative strains from each genotype (prototype, Long strain, JX198112; GA1, RSVA/Homo sapiens/USA/78I-004A-01-01/1977 strain, KU316106; GA2, HRSV/Yokohama.JPN/V13835/1996 strain, LC337817; GA3, HRSV/Yokohama.JPN/V10831/ 1992 strain, LC337812; GA4, RSVA/Homo sapiens/USA/81E-078-01/1977 strain, KU316149; GA5, RSVA/Homo sapiens/USA/MCRSV_259/1990 strain, MG642055; GA6, RSVA/Homo sapiens/USA/MCRSV_226/1982 strain, MG642063; GA7, RSVA/Homo sapiens/USA/84I-220A-01-01/1984 strain, KU316110; NA1, Kilifi_10028_12_ RSVA_2003 strain, KP317955) using MODELLER v9.20 [28]. The templates for homology modeling were based on the crystal structure of the protein (Protein Data Bank accession ID: 6EAD). Constructed models were minimized using GROMOS96 [29] implementation in the Swiss PDB Viewer v4.1 [30] and then evaluated by Ramachandran plots produced with Coot [31]. We analyzed conformational epitopes of the constructed models using DiscoTope 2.0 [32], BEpro [33], ElliPro [34], EPCES [35], and EPSVR [36] with cut-off values of −3.7 (DiscoTope 2.0), 1.3 (BEpro), 0.5 (ElliPro), and 70 (EPCES, EPSVR). The accuracy of the analyses was also supported among the consensus sites predicted by more than four of the five methods, and regions with close residues over two of the sites on the trimeric structure models were determined as conformational epitopes. We also estimated the amino acid substitution of the representative strains of each genotype from the prototype strain. Finally, we mapped predicted B-cell epitopes and amino acid substitution sites in each genotype and palivizumab epitopes on the models using Chimera v1.13.1 [37].

## 3. Results

### 3.1. Phylogenetic and Evolutionary Analyses of the RSV-A F Gene

**Figure 1:**
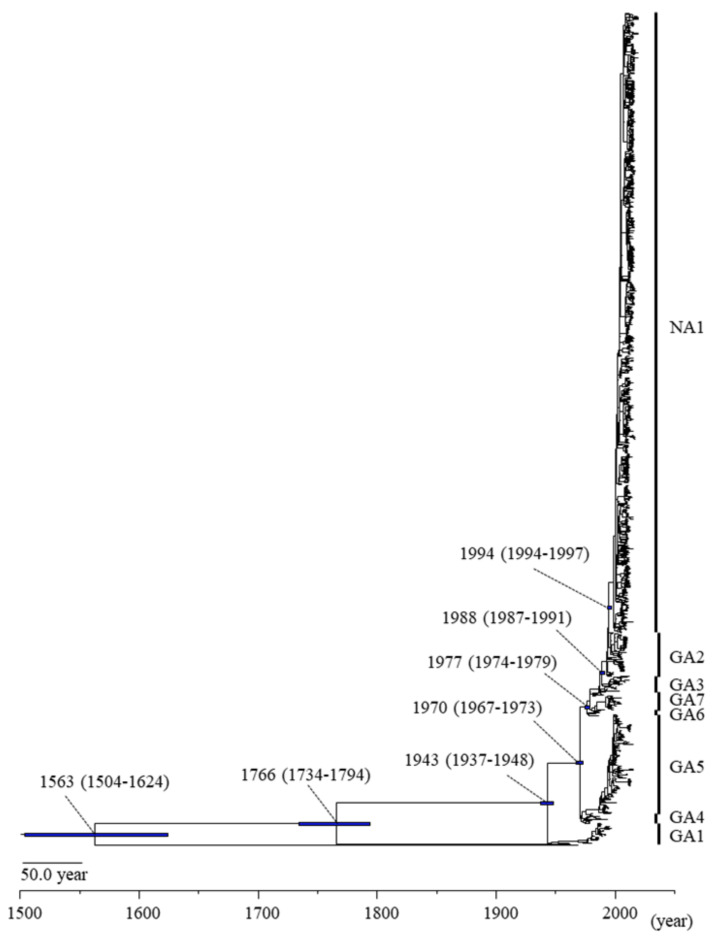
Time-scaled phylogenetic tree for the RSV F gene constructed using Bayesian Markov chain Monte Carlo method. The scale bar represents time (years). Blue bars indicate the 95% highest probability density (HPD) for a branched year.

At first, we performed a phylogenetic analysis using the MCMC method (Figure 1) for evaluating time scale evolution of the RSV-A F gene. As shown in Figure 1, human-RSV and bovine-RSV diverged from their common ancestor in 1563 (mean; 95% HPD, 1504–1624). RSV-A and -B diverged in 1766 (mean; 95% HPD, 1734–1794). RSV-A was classified into eight genotypes, with the most recently diverged genotype, NA1, accounting for 74.5% (1092/1465 strains) of the total. The respective divergence times and strain numbers of each genotype in RSV-A are shown in Table 1.

Secondly, we estimated the evolutionary rate of the RSV-A F gene. The evolutionary rate of the entire RSV-A was calculated to be 7.69 × 10^−4^ substitutions/site/year (95% HPD, 7.10–8.29 × 10^−4^ substitutions/site/year). The fastest evolutionary rate by genotype was 8.34 × 10^−4^ substitutions/site/year (95% HPD, 6.43 × 10^−4^–1.04 × 10^−3^ substitutions/site/year) for GA2, and the slowest was 4.48 × 10^−4^ substitutions/site/year (95% HPD, 2.79 × 10^−4^–6.24 × 10^−4^ substitutions/site/year) for GA1 (Supplementary Table S2). As noted, the genotype GA6 was not examined due to small strain numbers (six strains).

### 3.2. BSP Analyses

**Figure 2:**
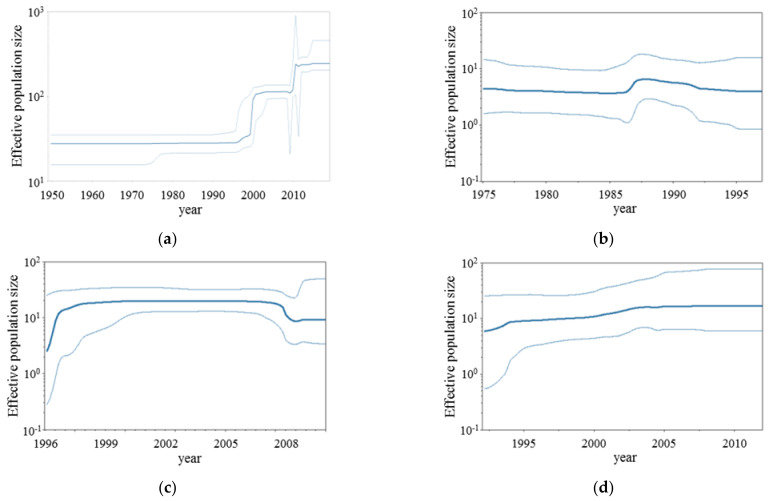

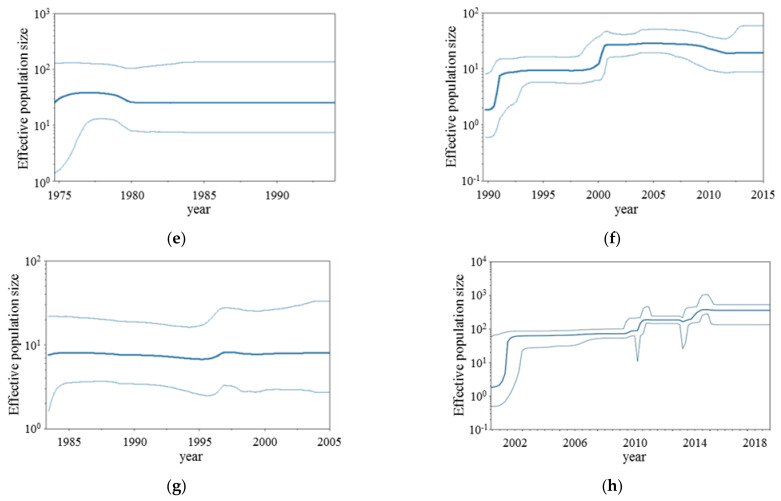
Bayesian skyline plots for the RSV-A F gene. Each panel illustrates the phylodynamics of all RSV-A strains (**a**), genotype GA1 (**b**), genotype GA2 (**c**), genotype GA3 (**d**), genotype GA4 (**e**), genotype GA5 (**f**), genotype GA7 (**g**), and genotype NA1 (**h**). The y-axis and x-axis indicate the effective population size and the time in years, respectively. The thick blue line shows the median value over time. 95% HPD intervals are represented in a thin blue line. As noted, the genotype GA6 was not examined due to small strain numbers (six strains).

We analyzed the phylodynamics of the present RSV-A strains using the Bayesian skyline plots method (Figure 2a–h). First, the effective population size of the present strains increased by two steps at around 2000 and 2010, respectively. These may reflect an increase in the strain numbers and diverged years of the genotypes GA5 and NA1 in the phylogenetic tree. The phylodynamics of each genotype exception of GA6 were also reflected in these factors.

### 3.3. Similarity Analysis and Phylogenetic Distances

**Figure 3:**
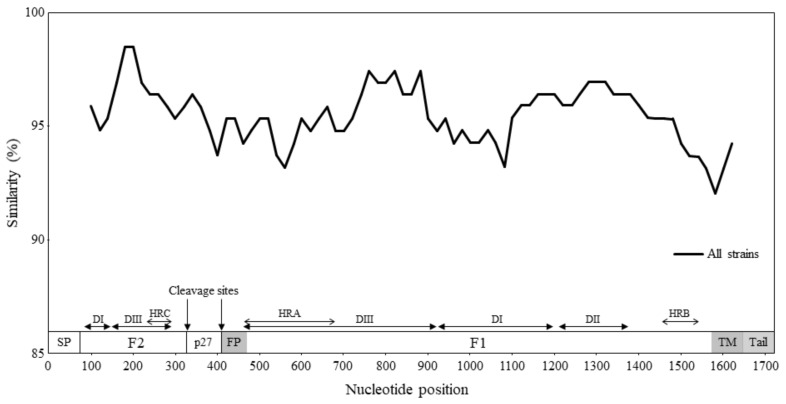
Similarity plot analysis of the F gene across all RSV-A strains. Nucleotide similarity to the prototype strain (Long strain, GenBank accession no. JX198112) was calculated using SimPlot analysis. Nucleotide position numbers correspond to the F gene in the prototype strain. The cleavage sites and the positions of each F1 and F2 subunits are shown below the graph [10]. SP, signal peptide; DI-DIII, domains I-III; HRA-HRC, heptad repeat A-C; p27, excised peptide; FP, fusion peptide; TM, transmembrane anchor; Tail, cytoplasmic tail.

**Figure 4:**
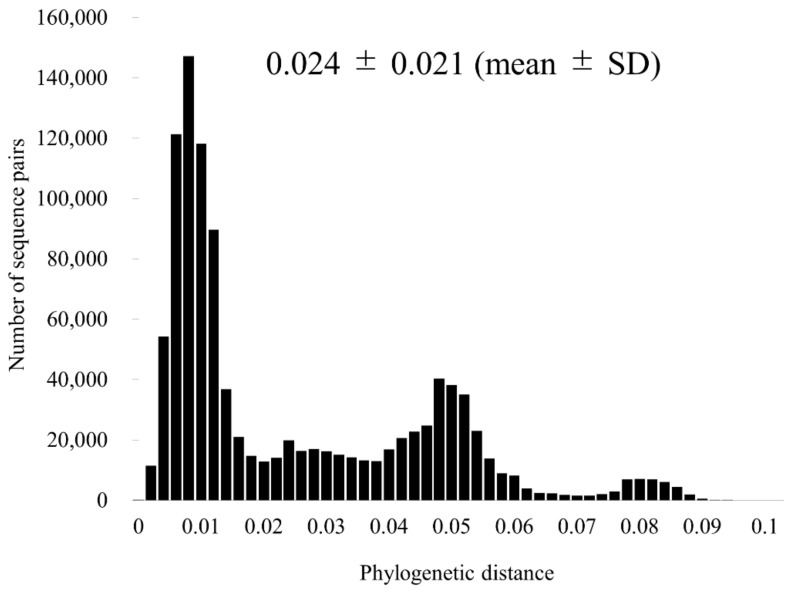
Distribution of the phylogenetic distances between the full-length sequences of the F gene of all RSV-A strains. The y-axis represents the number of sequence pairs corresponding to each distance. The x-axis shows phylogenetic distances.

Using SimPlot analysis, we examined the nucleotide similarity of the F gene of RSV-A (Figure 3). The results showed that the similarity of the entire RSV-A was high (>92%). Furthermore, we performed a phylogenetic distance analysis for the RSV-A F gene (Figure 4). Phylogenetic distance for the entire RSV-A was 0.024 ± 0.021 (mean ± SD). The most far apart phylogenetic distance was 0.017 ± 0.007 for GA3 by RSV-A genotypes; conversely, the closest was 0.006 ± 0.004 for GA1 (Supplementary Table S3).

### 3.4. Selective Pressure Analyses

We analyzed positive and negative selection sites of the F protein to determine selective pressure against the host. Unfortunately, there were no positive selection sites common to all four methods when calculated by SLAC, FEL, IFEL, and MEME. On the other hand, the negative selection sites were 165 for SLAC, 242 for FEL, and 176 for IFEL, and 137 sites were identified as common to all three methods.

### 3.5. Mapping of Amino Acid Substitution Sites and Conformational B-Cell Epitopes on the Structure of the RSV-A F Protein

**Figure 5:**
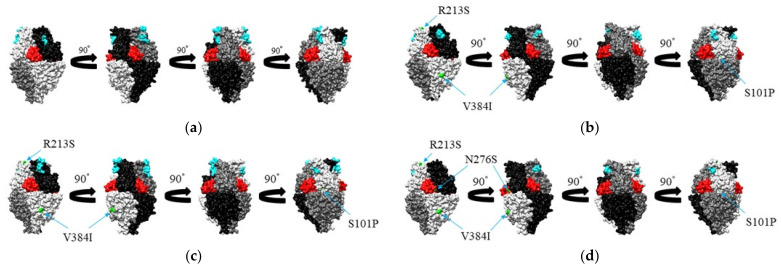
Structural models of the prefusion F protein of Long strain (**a**), genotype GA1 (**b**), genotype GA2 (**c**), and genotype NA1 (**d**). Chains of the trimeric structures are colored in light gray (chain A), dim gray (chain B), and black (chain C). Amino acid substitutions sites of chain A for each variant strain relative to the prototype strain are shown in green, and palivizumab epitopes are shown in red. The predicted conformational epitopes were indicated by cyan.

Structural models of the Long strain and representative strains of each genotype (NA1, GA1, GA2) were constructed (Figure 5). Since the amino acid sequences in the range included in the structural model of the representative strains of GA2, GA3, GA4, GA5, GA6, and GA7 were the same, only GA2 is shown. Then, amino acid substitutions corresponding to the prototype strain are shown in green. Three substitutions, “S101P,” “R213S,” and “V384I,” were detected in the amino acid sequence in the range included in the structural model of GA2 (Figure 5c). Four substitutions, “S101P,” “R213S,” “E356D,” and “V384I,” were found in the range of amino acid sequences included in the GA1 structural model (Figure 5b). E356D was not shown because it was almost hidden as an inner region of the protein structure. Likewise, four substitutions, “S101P,” “R213S,” “N276S,” and “V384I,” were present in the amino acid sequence in the range included in the structural model of NA1 (Figure 5d). We estimated the conformational B-cell epitope of RSV-A F protein (Table 2). Two epitopes were found in chain A, B, and C, respectively. Residues aa65~68 were in DIII of F2, and residues aa209~211 were in HRA. Both were located at the top of the structure protein models (colored in cyan).

## 4. Discussion

Molecular epidemiology of RSV infection based on the RSV-A F gene sequences has been studied in many reports [21,38,39,40,41]. However, most of these studies were domestic [35,36,37,38], while evolution, phylodynamics, and reinfection-related antigenicity of the virus based on the F gene are not exactly known. Therefore, we performed detailed evolutionary analyses of the RSV F gene using various in silico techniques combined with authentic bioinformatics to elucidate the evolution of the RSV-A F gene globally collected strains (1465 strains).

First, we constructed a time-scaled phylogenetic tree using MCMC methods (Figure 1). As a result of this tree, we demonstrated that the common ancestor of RSV-A, RSV-B, and bovine-RSV diverged around 450 years ago (1560s), and RSV-A and RSV-B diverged around 250 years ago (1760s). Moreover, the present RSV-A strains formed eight genotypes (GA1-GA7 and NA1) over 80 years. Interestingly, from the 1940s to the 1990s, seven genotypes (GA1-7) simultaneously emerged. Of these, off-springs of six genotypes (GA1, GA3-7) disappeared. As a result, an off-spring of the genotype GA2, i.e., genotype NA1, became a major prevalent genotype over the last 20 years. This genotype rapidly became dominant in the present phylogenetic tree. Between these values and the previous data, our report is different [21]. These may be partially due to the strain numbers and MCMC conditions [20]. Furthermore, we also estimated the evolutionary rates of the present strains. The evolutionary rate of the RSV-A F gene was estimated as 7.69 × 10^−4^ substitutions/site/year (s/s/y) (95% HPD, 7.10–8.29 × 10^−4^ s/s/y). This value was similar to the F gene of human respiro-virus type 3 [42]. Moreover, the rate was slower than that of the attachment glycoprotein (G) gene (another major antigen) [21].

Next, we assessed the phylodynamics of each genotype RSV-A F gene (excepting GA6), using the BSP method, during the past years. As a result, the genome population of genotypes GA1-3, GA5, and GA7 transiently increased from the 1980s to the 1990s. Moreover, genotype NA1 diverged from GA2 in 1994, and this became a recent prevalent genotype. Thus, these results regarding our phylodynamics data and epidemiological data may be compatible.

Next, to estimate the genetic divergence of the present strains, we calculated nucleotide identities and phylogenetic distances (Figure 4). The nucleotide identities among the strains were over 92%, and the ranges of phylogenetic distances were very narrow [0.024 ± 0.021 (mean ± SD)]. Thus, the results indicated that the F gene in these strains was highly conservative.

We also performed selective pressure analyses in the F protein genes among the strains. As a result, no selective pressure was estimated, while many negative selection sites (137 sites) were found. In general, positive selection sites may be shown to escape from the selective pressure in the host (i.e., immune system), while negative selection sites may act to prevent the protein deterioration [43]. These results suggested that the F protein was not sensitive to the selective pressure by the host, and amino acid substitutions of the proteins may maintain infectivity for the host cells.

Previous reports showed that the neutralization antibody (NT-Ab), including palivizumab (a monoclonal antibody to prevent RSV infection), could bind to the prefusion form of the F protein resulting in the prevention of RSV infection [11]. Conformational epitopes may act to produce the NT-Ab in the host [44]. Therefore, it is essential to estimate comparing conformational epitopes of viral antigens and NT-Ab binding sites [44]. If an incompatibility is seen in them, infections may recur. Therefore, we estimated comparing conformational epitopes and NT-Ab binding sites (palivizumab binding sites) in the prefusion type of the F protein (Figure 5 and Table 2). As a result, conformational epitopes did not correspond to the NT-Ab binding sites in the prefusion type of the protein. The results suggested that this mismatch may be partially responsible for RSV reinfection and another virus such as human respiro-virus type 3 [42]. Furthermore, an amino acid substitution of N276S in the palivizumab binding sites may reduce this drug effect. Our results showed that almost all strains (94.4%) of the recent prevalent genotype (NA1) had the N276S. Thus, most prevalent RSV-A strains had partial resistance to the palivizumab [45]. Presently, palivizumab is only available to prevent severe RSV infection in infants with underlying conditions such as congenital heart/lung diseases, low birth infants, and Down’s syndrome [46]. Continuous virus surveillance regarding the RSV-A F gene sequences may be needed to monitor drug sensitivity against RSV-A. Moreover, the NT-Ab binds to prefusion F protein and can inhibit infection to the host cell. In contrast, the antibody-bound post-fusion F protein has no affect [12]. Our previous report showed relationships between NT-Ab binding sites and conformational epitopes in the post-fusion F protein [21]. Thus, the current new data may be more precise than the previous data [21]. Moreover, there is regional bias of sequence availability in GenBank. This is a limitation of the present study.

## 5. Conclusions

We performed detailed evolutionary analyses of respiratory syncytial virus subgroup A (RSV-A) fusion (F) gene in the globally collected strains using various bioinformatics methods. A time-scaled evolutionary tree showed that a common ancestor of RSV-A, RSV-B, and bovine-RSV diverged around 450 years ago, and RSV-A and RSV-B diverged around 250 years ago. The RSV-A F gene formed eight genotypes (GA1-GA7 and NA1) during the last 80 years. Phylodynamics of RSV-A F gene including all genotype strains increased twice tin the 1990s and 2010s, while patterns of each RSV-A genotype were distinct. Phylogenetic distance analysis suggested that the genetic distances of the strains were short. No positive selection sites were found, while many negative selection sites were estimated. Moreover, the F protein 3D structure and conformational epitope analyses showed that the conformational epitopes did not correspond to the NT-Ab binding sites of the F protein. These data implied that the RSV-A F gene is relatively conserved. Mismatches between conformational epitopes and NT-Ab binding sites of the F protein may be responsible for virus reinfection.

## Figures and Tables

**Table 1:** The divergence year of RSV-A genotypes.

Virus	Genotype	Diverged Year (95%HPD)	Strain Numbers
RSV-A	GA1	1943 (1937–1948)	35
	GA4	1970 (1967–1973)	17
	GA5	1975 (1973–1977)	174
	GA6	1977 (1974–1979)	6
	GA7	1977 (1974–1979)	34
	GA3	1979 (1978–1981)	28
	GA2	1988 (1987–1991)	77
	NA1	1994 (1994–1997)	1092
RSV-B		1766 (1734–1794)	1
Bovine-RSV		1563 (1504–1624)	1

**Table 2:** Predicted conformational epitopes and amino acid residues corresponding to the prototype strain (Long strain).

Chain		A						B							C						
Residue		65	66	67	68	210	211	65	66	67	68	209	210	211	65	66	67	68	209	210	211
Prototype strain		K	E	N	K	Q	S	K	E	N	K	K	Q	S	K	E	N	K	K	Q	S
Genotype	NA1	.	.	.	.	.	.	.	.	.	.	.	.	.	.	.	.	.	.	.	.
	GA1	.	.	.	.	.	.	.	.	.	.	.	.	.	.	.	.	.	.	.	.
	GA2	.	.	.	.	.	.	.	.	.	.	.	.	.	.	.	.	.	.	.	.
	GA3	.	.	.	.	.	.	.	.	.	.	.	.	.	.	.	.	.	.	.	.
	GA4	.	.	.	.	.	.	.	.	.	.	.	.	.	.	.	.	.	.	.	.
	GA5	.	.	.	.	.	.	.	.	.	.	.	.	.	.	.	.	.	.	.	.
	GA6	.	.	.	.	.	.	.	.	.	.	.	.	.	.	.	.	.	.	.	.
	GA7	.	.	.	.	.	.	.	.	.	.	.	.	.	.	.	.	.	.	.	.

Shaded regions reflect the putative epitopes of each genotype. Amino acid residues of each genotype identical to the prototype strain are indicated by dots.

## Supplementary Materials

The following are available online at https://www.mdpi.com/article/10.3390/v13122525/s1, Table S1: Strains used in this study, Table S2: Evolutionary rates for each genotype, Table S3: Phylogenetic distances for each genotype.
