# Supplementary material for: Detailed Evolutionary Analyses of the F Gene in the Respiratory Syncytial Virus Subgroup A
Source: Viruses. 2021 Dec 15;13(12):2525. doi: 10.3390/v13122525 (PMC8706373; doi:10.3390/v13122525)
Supplement: Supplementary file 1 [file viruses-13-02525-s001.zip › viruses-1476447 - supplementary.pdf]

viruses

MDPI

Supplementary Data

Supplementary Tables

Supplementary Table S1. Strains used in this study.

GenBank accession No.	Strain	Collection year	Country	Genotype	References or authorship
JX198113	Tracy	1987	USA	GA1	1
KJ723467	RSVA/Homo sapiens/USA/86I-044A-01/1986	1986	USA	GA1	Das, S.R. et al.
KJ723474	RSVA/Homo sapiens/USA/89I-123A-01/1989	1989	USA	GA1	Das, S.R. et al.
KJ723475	RSVA/Homo sapiens/USA/86I-139A-01/1986	1986	USA	GA1	Das, S.R. et al.
KJ723478	RSVA/Homo sapiens/USA/82I-158A-01/1982	1982	USA	GA1	Das, S.R. et al.
KJ723489	RSVA/Homo sapiens/USA/90I-283A-01/1990	1990	USA	GA1	Das, S.R. et al.
KP258695	RSVA/Homo sapiens/USA/88E-072-01/1988	1988	USA	GA1	Das, S.R. et al.
KP258717	RSVA/Homo sapiens/USA/94E-091-01/1994	1994	USA	GA1	Das, S.R. et al.
KP258719	RSVA/Homo sapiens/USA/88E-082-01/1988	1988	USA	GA1	Das, S.R. et al.
KP258729	RSVA/Homo sapiens/USA/90P-147-01/1990	1990	USA	GA1	Das, S.R. et al.
KP258741	RSVA/Homo sapiens/USA/90E-112-01/1990	1990	USA	GA1	Das, S.R. et al.
KP258744	RSVA/Homo sapiens/USA/95P-073-01/1995	1995	USA	GA1	Das, S.R. et al.
KU316103	RSVA/Homo sapiens/USA/83G-012-01/1983	1983	USA	GA1	Das, S.R. et al.
KU316106	RSVA/Homo sapiens/USA/78I-004A-01-01/1977	1977	USA	GA1	Das, S.R. et al.
KU316107	RSVA/Homo sapiens/USA/95P-071-01/1995	1995	USA	GA1	Das, S.R. et al.
KU316109	RSVA/Homo sapiens/USA/92I-109A-01-01/1992	1992	USA	GA1	Das, S.R. et al.
KU316119	RSVA/Homo sapiens/USA/89E-503-01/1989	1989	USA	GA1	Das, S.R. et al.
KU316120	RSVA/Homo sapiens/USA/86I-024A-01-01/1985	1985	USA	GA1	Das, S.R. et al.
KU316123	RSVA/Homo sapiens/USA/97I-058A-01-01/1997	1997	USA	GA1	Das, S.R. et al.
KU316124	RSVA/Homo sapiens/USA/92I-050A-01-01/1992	1992	USA	GA1	Das, S.R. et al.
KU316140	RSVA/Homo sapiens/USA/78I-046A-01-01/1978	1978	USA	GA1	Das, S.R. et al.
KU316146	RSVA/Homo sapiens/USA/83G-002-01/1983	1983	USA	GA1	Das, S.R. et al.
KU316152	RSVA/Homo sapiens/USA/85P-399-01/1985	1985	USA	GA1	Das, S.R. et al.
KU316153	RSVA/Homo sapiens/USA/88E-042-01/1988	1988	USA	GA1	Das, S.R. et al.
KU316162	RSVA/Homo sapiens/USA/88E-033-01/1988	1988	USA	GA1	Das, S.R. et al.
KU316164	RSVA/Homo sapiens/USA/95P-326-01/1995	1995	USA	GA1	Das, S.R. et al.
KU316165	RSVA/Homo sapiens/USA/88I-141A-01-01/1987	1987	USA	GA1	Das, S.R. et al.
KU316168	RSVA/Homo sapiens/USA/78I-026A-01-01/1977	1977	USA	GA1	Das, S.R. et al.
KU316171	RSVA/Homo sapiens/USA/78I-016A-01-01/1977	1977	USA	GA1	Das, S.R. et al.
KU316174	RSVA/Homo sapiens/USA/88I-095A-01-01/1987	1987	USA	GA1	Das, S.R. et al.
MF361904	MON/9/92	1992	Uruguay	GA1	2
MG642060	RSVA/Homo sapiens/USA/MCRSV_211/1980	1980	USA	GA1	Shabman, R. et al.
MG642067	RSVA/Homo sapiens/USA/MCRSV_254/1987	1987	USA	GA1	Shabman, R. et al.
MG642075	RSVA/Homo sapiens/USA/MCRSV_248/1986	1986	USA	GA1	Shabman, R. et al.
MG813989	A-TX-Tracy-1989-WGS	1989	USA	GA1	Piedra, F.-A. et al.
JF920057	A/WI/629-4239/98	1998	USA	GA2	3
JQ901447	01-000312	2001	Netherlands	GA2	4
JQ901452	01-031282	2001	Netherlands	GA2	4
JQ901453	02-000110	2002	Netherlands	GA2	4
JQ901457	03-036456	2003	Netherlands	GA2	4
JQ901458	03-036544	2003	Netherlands	GA2	4
JX015480	07-040054	2007	Netherlands	GA2	4
JX015486	05-000417	2005	Netherlands	GA2	4

JX069799	RSVA/human/USA/A2001-03-12/2001/GA2	2001	USA	GA2	Lorenzi, H. et al.
JX198105	TX-79309	2005	USA	GA2	1
JX198108	TX-79321	2005	USA	GA2	1
JX198110	TX-79334	2005	USA	GA2	1
JX198120	TX-79223	2004	USA	GA2	1
JX198126	TX-79256	2004	USA	GA2	1
JX198128	TX-79258	2004	USA	GA2	1
JX198131	TX-79299	2005	USA	GA2	1
JX198134	TX-79308	2005	USA	GA2	1
KF826848	RSVA/Homo sapiens/AUS/249/2007	2007	Australia	GA2	Lorenzi, H. et al.

Supplementary Table S1 (continued). Strains used in this study.

GenBank accession No.	Strain	Collection year	Country	Genotype	References or authorship
KJ627248	RSVA/Homo sapiens/PER/FLA6927/2009	2009	Peru	GA2	Wentworth, D.E. et al.
KJ627255	RSVA/Homo sapiens/PER/FLA6533/2009	2009	Peru	GA2	Wentworth, D.E. et al.
KJ627260	RSVA/Homo sapiens/PER/FLA0756/2008	2008	Peru	GA2	Wentworth, D.E. et al.
KJ627276	RSVA/Homo sapiens/PER/FLA6891/2009	2009	Peru	GA2	Wentworth, D.E. et al.
KJ627284	RSVA/Homo sapiens/PER/FLA7779/2009	2009	Peru	GA2	Wentworth, D.E. et al.
KJ627289	RSVA/Homo sapiens/PER/FLA7440/2009	2009	Peru	GA2	Wentworth, D.E. et al.
KJ627305	RSVA/Homo sapiens/PER/FLU5848/2007	2007	Peru	GA2	Wentworth, D.E. et al.
KJ627311	RSVA/Homo sapiens/PER/FLA2268/2008	2008	Peru	GA2	Wentworth, D.E. et al.
KJ627314	RSVA/Homo sapiens/PER/FLA7464/2009	2009	Peru	GA2	Wentworth, D.E. et al.
KJ627324	RSVA/Homo sapiens/PER/FLA6757/2009	2009	Peru	GA2	Wentworth, D.E. et al.
KJ627327	RSVA/Homo sapiens/PER/FLU7450/2007	2007	Peru	GA2	Wentworth, D.E. et al.
KJ627336	RSVA/Homo sapiens/PER/FLA0535/2008	2008	Peru	GA2	Wentworth, D.E. et al.
KJ627339	RSVA/Homo sapiens/PER/FLA6331/2009	2009	Peru	GA2	Wentworth, D.E. et al.
KJ627352	RSVA/Homo sapiens/PER/FLU5725/2007	2007	Peru	GA2	Wentworth, D.E. et al.
KJ627353	RSVA/Homo sapiens/PER/FLA0489/2008	2008	Peru	GA2	Wentworth, D.E. et al.
KJ627361	RSVA/Homo sapiens/PER/FLU5529/2007	2007	Peru	GA2	Wentworth, D.E. et al.
KJ627366	RSVA/Homo sapiens/PER/FLA6758/2009	2009	Peru	GA2	Wentworth, D.E. et al.
KP317933	Kilifi_10891_51_RSVA_2008	2008	Kenya	GA2	5
KP317937	Kilifi_10891_59_RSVA_2007	2007	Kenya	GA2	5
KP317942	Kilifi_10891_57_RSVA_2006	2006	Kenya	GA2	5
KP317944	Kilifi_9696_45_RSVA_2006	2006	Kenya	GA2	5
KP317950	Kilifi_10899_38_RSVA_2009	2009	Kenya	GA2	5
KP856968	RSVA/Homo sapiens/USA/98I-003A-01/1998	1998	USA	GA2	Das, S.R. et al.
KU316091	RSVA/Homo sapiens/USA/98I-005A-01-01/1998	1998	USA	GA2	Das, S.R. et al.
KU316141	RSVA/Homo sapiens/USA/98E-126-01/1998	1998	USA	GA2	Das, S.R. et al.
LC337816	HRSV/Yokohama.JPN/V13828/1996	1996	Japan	GA2	Saikusa, M. et al.
LC337817	HRSV/Yokohama.JPN/V13835/1996	1996	Japan	GA2	Saikusa, M. et al.
LC337818	HRSV/Yokohama.JPN/V13933/1997	1997	Japan	GA2	Saikusa, M. et al.
LC337820	HRSV/Yokohama.JPN/V14040/1997	1997	Japan	GA2	Saikusa, M. et al.
LC337821	HRSV/Yokohama.JPN/V14140/1997	1997	Japan	GA2	Saikusa, M. et al.
LC337823	HRSV/Yokohama.JPN/V14179/1997	1997	Japan	GA2	Saikusa, M. et al.
LC337833	HRSV/Yokohama.JPN/V14835/1997	1997	Japan	GA2	Saikusa, M. et al.
LC337842	HRSV/Yokohama.JPN/P203/1999	1999	Japan	GA2	Saikusa, M. et al.
LC337892	HRSV/Yokohama.JPN/P2363/2003	2003	Japan	GA2	Saikusa, M. et al.
LC337914	HRSV/Yokohama.JPN/P3200/2005	2005	Japan	GA2	Saikusa, M. et al.
LC337917	HRSV/Yokohama.JPN/P3587/2006	2006	Japan	GA2	Saikusa, M. et al.
LC337918	HRSV/Yokohama.JPN/P3623/2006	2006	Japan	GA2	Saikusa, M. et al.
LC337919	HRSV/Yokohama.JPN/P3688/2007	2007	Japan	GA2	Saikusa, M. et al.
LC365787	HRSV/Yokohama.JPN/P3941/2007	2007	Japan	GA2	Saikusa, M. et al.
LC365789	HRSV/Yokohama.JPN/P4073/2007	2007	Japan	GA2	Saikusa, M. et al.
LC365792	HRSV/Yokohama.JPN/P4101/2007	2007	Japan	GA2	Saikusa, M. et al.
LC365795	HRSV/Yokohama.JPN/P4388/2008	2008	Japan	GA2	Saikusa, M. et al.
LC365797	HRSV/Yokohama.JPN/P4403/2008	2008	Japan	GA2	Saikusa, M. et al.
LC365799	HRSV/Yokohama.JPN/P4422/2008	2008	Japan	GA2	Saikusa, M. et al.
LC365801	HRSV/Yokohama.JPN/P4427/2008	2008	Japan	GA2	Saikusa, M. et al.
LC365803	HRSV/Yokohama.JPN/P4432/2008	2008	Japan	GA2	Saikusa, M. et al.
LC365815	HRSV/Yokohama.JPN/P4487/2008	2008	Japan	GA2	Saikusa, M. et al.
LC365816	HRSV/Yokohama.JPN/P4494/2008	2008	Japan	GA2	Saikusa, M. et al.
MF445965	FJ081103-01	2008	China	GA2	Su, Y. et al.

MF445966	FJ090104-33	2009	China	GA2	Su, Y. et al.
MF445967	FJ090302-03	2009	China	GA2	Su, Y. et al.
MF445968	FJ101224-40	2010	China	GA2	Su, Y. et al.
MF445969	FJ101118-01	2010	China	GA2	Su, Y. et al.
MF445970	FJ101225-02	2010	China	GA2	Su, Y. et al.
MF445971	FJ101011-37	2010	China	GA2	Su, Y. et al.
MF445972	FJ101011-38	2010	China	GA2	Su, Y. et al.
MF445973	FJ101213-49	2010	China	GA2	Su, Y. et al.
MF445974	FJ091113-21A	2009	China	GA2	Su, Y. et al.
MF445975	FJ091221-01A	2009	China	GA2	Su, Y. et al.

Supplementary Table S1 (continued). Strains used in this study.

GenBank accession No.	Strain	Collection year	Country	Genotype	References or authorship
FJ948820	98-25147-X	1999	Netherlands	GA3	6
JX069798	RSVA/human/USA/A2001-02-20/2001/GA2	2001	USA	GA3	Lorenzi, H. et al.
JX682716	CQ_Mar-2012/3155	2012	China	GA3	7
JX682745	CQ_Dec-2010/1148	2010	China	GA3	7
KJ627647	RSV-A/US/BID-V8313/2001	2001	USA	GA3	Newman, R.M. et al.
KJ627681	RSV-A/US/BID-V8425/2001	2001	USA	GA3	Newman, R.M. et al.
KP119746	HRSV-A-GZ08-12	2012	Hong Kong	GA3	8
KP218910	HRSV-A-GZ08-0	2008	China	GA3	8
KU316118	RSVA/Homo sapiens/USA/96I-029A-01-01/1996	1996	USA	GA3	Das, S.R. et al.
KU316139	RSVA/Homo sapiens/USA/94I-091A-01-01/1994	1994	USA	GA3	Das, S.R. et al.
KU950573	RSVA/Homo sapiens/USA/TH_10681/2006	2006	USA	GA3	Das, S.R. et al.
KY296708	BJ04-01	2004	China	GA3	9
KY296709	BJ04-23	2004	China	GA3	9
KY296710	BJ04-32	2004	China	GA3	9
KY296711	BJ04-34	2004	China	GA3	9
KY296712	BJ04-44	2004	China	GA3	9
KY296715	BJ_09-105	2005	China	GA3	9
KY296718	BJ_10-02	2005	China	GA3	9
KY296747	GZ11-18	2005	China	GA3	9
KY296751	GZ11-21	2005	China	GA3	9
LC337812	HRSV/Yokohama.JPN/V10831/1992	1992	Japan	GA3	Saikusa, M. et al.
LC337819	HRSV/Yokohama.JPN/V13968/1997	1997	Japan	GA3	Saikusa, M. et al.
LC337824	HRSV/Yokohama.JPN/V14337/1997	1997	Japan	GA3	Saikusa, M. et al.
LC337830	HRSV/Yokohama.JPN/V14823/1997	1997	Japan	GA3	Saikusa, M. et al.
LC337875	HRSV/Yokohama.JPN/P1419/2001	2001	Japan	GA3	Saikusa, M. et al.
LC337897	HRSV/Yokohama.JPN/P2746/2004	2004	Japan	GA3	Saikusa, M. et al.
MG642033	RSVA/Homo sapiens/USA/MCRSV_265/1994	1994	USA	GA3	Shabman, R. et al.
MG642050	RSVA/Homo sapiens/USA/MCRSV_261/1994	1994	USA	GA3	Shabman, R. et al.
JX198137	TX-37425	1987	USA	GA4	1
KJ723486	RSVA/Homo sapiens/USA/79I-039A-01/1979	1979	USA	GA4	Das, S.R. et al.
KJ723488	RSVA/Homo sapiens/USA/81I-123A-01/1981	1981	USA	GA4	Das, S.R. et al.
KP258696	RSVA/Homo sapiens/USA/86E-155-01/1986	1986	USA	GA4	Das, S.R. et al.
KP258704	RSVA/Homo sapiens/USA/94I-009A-01/1994	1994	USA	GA4	Das, S.R. et al.
KP258715	RSVA/Homo sapiens/USA/88E-009-01/1988	1988	USA	GA4	Das, S.R. et al.
KP856969	RSVA/Homo sapiens/USA/79E-495-01/1979	1979	USA	GA4	Das, S.R. et al.
KU316135	RSVA/Homo sapiens/USA/81I-090A-01-01/1981	1981	USA	GA4	Das, S.R. et al.
KU316137	RSVA/Homo sapiens/USA/79E-040-01/1979	1979	USA	GA4	Das, S.R. et al.
KU316143	RSVA/Homo sapiens/USA/82G-164-01/1982	1982	USA	GA4	Das, S.R. et al.
KU316149	RSVA/Homo sapiens/USA/81E-078-01/1977	1977	USA	GA4	Das, S.R. et al.
KU316150	RSVA/Homo sapiens/USA/78G-168-01/1978	1978	USA	GA4	Das, S.R. et al.
KU316157	RSVA/Homo sapiens/USA/79I-050A-01-01/1979	1979	USA	GA4	Das, S.R. et al.
KU316178	RSVA/Homo sapiens/USA/79E-191-01/1979	1979	USA	GA4	Das, S.R. et al.
MG642028	RSVA/Homo sapiens/USA/MCRSV_220/1980	1980	USA	GA4	Shabman, R. et al.
MG642040	RSVA/Homo sapiens/USA/MCRSV_214/1980	1980	USA	GA4	Shabman, R. et al.
MG642074	RSVA/Homo sapiens/USA/MCRSV_209/1980	1980	USA	GA4	Shabman, R. et al.
JQ901448	01-000583	2001	Netherlands	GA5	4
JQ901449	01-000868	2001	Netherlands	GA5	4
JQ901450	01-002215	2001	Netherlands	GA5	4
JQ901451	01-002279	2001	Netherlands	GA5	4

JQ901454	02-00291	2002	Netherlands	GA5	4
JQ901455	02-017863	2002	Netherlands	GA5	4
JQ901456	03-033338	2003	Netherlands	GA5	4
JX015485	05-000257	2005	Netherlands	GA5	4
JX015487	06-000103	2006	Netherlands	GA5	4
JX015488	06-000827	2006	Netherlands	GA5	4
JX069803	RSVA/human/USA/A2000-03-04/2000	2000	USA	GA5	Lorenzi, H. et al.
JX198106	TX-79310	2005	USA	GA5	1
JX198109	TX-79326	2005	USA	GA5	1
JX198111	TX-79365	2005	USA	GA5	1

Supplementary Table S1 (continued). Strains used in this study.

GenBank accession No.	Strain	Collection year	Country	Genotype	References or authorship
JX198114	TX-50106	1991	USA	GA5	1
JX198115	TX-50437	1991	USA	GA5	1
JX198116	TX-61245	1993	USA	GA5	1
JX198117	TX-79216	2004	USA	GA5	1
JX198118	TX-79218	2004	USA	GA5	1
JX198119	TX-79219	2004	USA	GA5	1
JX198121	TX-79228	2004	USA	GA5	1
JX198122	TX-79230	2004	USA	GA5	1
JX198123	TX-79240	2004	USA	GA5	1
JX198124	TX-79248	2004	USA	GA5	1
JX198125	TX-79254	2004	USA	GA5	1
JX198127	TX-79257	2004	USA	GA5	1
JX198129	TX-79285	2004	USA	GA5	1
JX198133	TX-79306	2005	USA	GA5	1
JX198135	TX-68357	1994	USA	GA5	1
JX198139	TX-66125	1994	USA	GA5	1
KC618407	9278	2007	Italy	GA5	Piralla, A. et al.
KF530260	RSVA/Homo sapiens/ARG/170/2005	2005	Argentina	GA5	Lorenzi, H. et al.
KF530268	RSVA/Homo sapiens/MEX/59/2007	2007	Mexico	GA5	Lorenzi, H. et al.
KF826816	RSVA/Homo sapiens/MEX/25/2005	2005	Mexico	GA5	Lorenzi, H. et al.
KF826823	RSVA/Homo sapiens/USA/629-4360/1998	1998	USA	GA5	Lorenzi, H. et al.
KF826824	RSVA/Homo sapiens/USA/629-4392/1998	1998	USA	GA5	Lorenzi, H. et al.
KF826826	RSVA/Homo sapiens/MEX/23/2004	2004	Mexico	GA5	Lorenzi, H. et al.
KF826827	RSVA/Homo sapiens/ARG/159/2004	2004	Argentina	GA5	Lorenzi, H. et al.
KF826828	RSVA/Homo sapiens/ARG/162/2004	2004	Argentina	GA5	Lorenzi, H. et al.
KF826832	RSVA/Homo sapiens/ITA/120/2009	2009	Italy	GA5	Lorenzi, H. et al.
KF826836	RSVA/Homo sapiens/MEX/26/2006	2006	Mexico	GA5	Lorenzi, H. et al.
KF826837	RSVA/Homo sapiens/MEX/27/2006	2006	Mexico	GA5	Lorenzi, H. et al.
KF826841	RSVA/Homo sapiens/ARG/190/2007	2007	Argentina	GA5	Lorenzi, H. et al.
KF826846	RSVA/Homo sapiens/ARG/202/2008	2008	Argentina	GA5	Lorenzi, H. et al.
KF826847	RSVA/Homo sapiens/AUS/248/2007	2007	Australia	GA5	Lorenzi, H. et al.
KF826850	RSVA/Homo sapiens/USA/629-11-1/2008	2008	USA	GA5	Lorenzi, H. et al.
KF826852	RSVA/Homo sapiens/USA/629-1/2007	2007	USA	GA5	Lorenzi, H. et al.
KF826854	RSVA/Homo sapiens/ITA/119/2009	2009	Italy	GA5	Lorenzi, H. et al.
KF973332	RSV-A/US/BID-V7349/2002	2002	USA	GA5	10
KF973340	RSV-A/US/BID-V7359/2002	2002	USA	GA5	10
KJ627649	RSV-A/US/BID-V8316/2001	2001	USA	GA5	Newman, R.M. et al.
KJ627652	RSV-A/US/BID-V8320/2002	2002	USA	GA5	Newman, R.M. et al.
KJ627654	RSV-A/US/BID-V8323/2002	2002	USA	GA5	Newman, R.M. et al.
KJ627656	RSV-A/US/BID-V8352/2003	2003	USA	GA5	Newman, R.M. et al.
KJ627662	RSV-A/US/BID-V8372/2003	2003	USA	GA5	Newman, R.M. et al.
KJ627664	RSV-A/US/BID-V8376/2003	2003	USA	GA5	Newman, R.M. et al.
KJ627668	RSV-A/US/BID-V8384/2003	2003	USA	GA5	Newman, R.M. et al.
KJ627671	RSV-A/US/BID-V8405/2003	2003	USA	GA5	Newman, R.M. et al.
KJ627675	RSV-A/US/BID-V8415/2001	2001	USA	GA5	Newman, R.M. et al.
KJ627676	RSV-A/US/BID-V8419/2001	2001	USA	GA5	Newman, R.M. et al.
KJ627679	RSV-A/US/BID-V8423/2001	2001	USA	GA5	Newman, R.M. et al.
KJ627684	RSV-A/US/BID-V8434/2001	2001	USA	GA5	Newman, R.M. et al.
KJ627687	RSV-A/US/BID-V8437/2001	2001	USA	GA5	Newman, R.M. et al.

KJ627690	RSV-A/US/BID-V8456/2001	2001	USA	GA5	Newman, R.M. et al.
KJ627693	RSV-A/US/BID-V8466/2001	2001	USA	GA5	Newman, R.M. et al.
KJ627696	RSV-A/US/BID-V8471/2001	2001	USA	GA5	Newman, R.M. et al.
KJ627701	RSV-A/US/BID-V8477/2001	2001	USA	GA5	Newman, R.M. et al.
KJ627707	RSV-A/US/BID-V8486/2001	2001	USA	GA5	Newman, R.M. et al.
KJ627709	RSV-A/US/BID-V8491/2001	2001	USA	GA5	Newman, R.M. et al.
KJ627710	RSV-A/US/BID-V8493/2001	2001	USA	GA5	Newman, R.M. et al.
KJ627717	RSV-A/US/BID-V8509/2003	2001	USA	GA5	Newman, R.M. et al.
KJ627722	RSV-A/US/BID-V8523/2004	2004	USA	GA5	Newman, R.M. et al.
KJ627725	RSV-A/US/BID-V8526/2004	2004	USA	GA5	Newman, R.M. et al.

Supplementary Table S1 (continued). Strains used in this study.

GenBank accession No.	Strain	Collection year	Country	Genotype	References or authorship
KJ627729	RSV-A/US/BID-V8537/2003	2003	USA	GA5	Newman, R.M. et al.
KJ627731	RSV-A/US/BID-V8540/2003	2003	USA	GA5	Newman, R.M. et al.
KJ627733	RSV-A/US/BID-V8547/2003	2003	USA	GA5	Newman, R.M. et al.
KJ641590	RSV-A/US/BID-V9458/2012	2012	USA	GA5	Newman, R.M. et al.
KJ643492	RSV-A/US/BID-V9411/2013	2013	USA	GA5	Newman, R.M. et al.
KJ643493	RSV-A/US/BID-V9412/2013	2013	USA	GA5	Newman, R.M. et al.
KJ643498	RSV-A/US/BID-V9419/2013	2013	USA	GA5	Newman, R.M. et al.
KJ643504	RSV-A/US/BID-V9427/2013	2013	USA	GA5	Newman, R.M. et al.
KJ643529	RSV-A/US/BID-V9463/2013	2013	USA	GA5	Newman, R.M. et al.
KJ643548	RSV-A/US/BID-V9489/2013	2013	USA	GA5	Newman, R.M. et al.
KJ643560	RSV-A/US/BID-V9507/2013	2013	USA	GA5	Newman, R.M. et al.
KJ643561	RSV-A/US/BID-V9510/2013	2013	USA	GA5	Newman, R.M. et al.
KJ643585	RSV-A/US/BID-V9553/2013	2013	USA	GA5	Newman, R.M. et al.
KJ643586	RSV-A/US/BID-V9554/2013	2013	USA	GA5	Newman, R.M. et al.
KJ672462	RSVA/Homo sapiens/USA/LA2_84/2013	2013	USA	GA5	Das, S. et al.
KJ672474	RSVA/Homo sapiens/USA/LA2_38/2012	2012	USA	GA5	Das, S. et al.
KJ672479	RSVA/Homo sapiens/USA/LA2_18/2013	2013	USA	GA5	Das, S. et al.
KJ672483	RSVA/Homo sapiens/USA/LA2_19/2013	2013	USA	GA5	Das, S. et al.
KJ723462	RSVA/Homo sapiens/USA/92I-068A-01/1992	1992	USA	GA5	Das, S. et al.
KJ723465	RSVA/Homo sapiens/USA/92I-094A-01/1992	1992	USA	GA5	Das, S. et al.
KJ723473	RSVA/Homo sapiens/USA/90I-229A-01/1990	1990	USA	GA5	Das, S. et al.
KJ723490	RSVA/Homo sapiens/USA/92I-048A-01/1992	1992	USA	GA5	Das, S. et al.
KJ939943	VN-360-6/10	2010	Viet Nam	GA5	11
KJ939948	VN-391-7/10	2010	Viet Nam	GA5	11
KP258701	RSVA/Homo sapiens/USA/94E-014-01/1994	1994	USA	GA5	Das, S.R. et al.
KP258703	RSVA/Homo sapiens/USA/97I-009A-01/1997	1997	USA	GA5	Das, S.R. et al.
KP258707	RSVA/Homo sapiens/USA/95P-047-01/1995	1995	USA	GA5	Das, S.R. et al.
KP258710	RSVA/Homo sapiens/USA/91E-611-01/1991	1991	USA	GA5	Das, S.R. et al.
KP258722	RSVA/Homo sapiens/USA/93E-014-01/1993	1993	USA	GA5	Das, S.R. et al.
KP258726	RSVA/Homo sapiens/USA/96E-128-01/1996	1996	USA	GA5	Das, S.R. et al.
KP258727	RSVA/Homo sapiens/USA/97E-101-01/1997	1997	USA	GA5	Das, S.R. et al.
KP258728	RSVA/Homo sapiens/USA/92E-024-01/1992	1992	USA	GA5	Das, S.R. et al.
KP258732	RSVA/Homo sapiens/USA/91E-597-01/1991	1991	USA	GA5	Das, S.R. et al.
KP258740	RSVA/Homo sapiens/USA/93E-065-01/1993	1993	USA	GA5	Das, S.R. et al.
KP317949	Kilifi_11866_65_RSVA_2003	2003	Kenya	GA5	5
KP317956	Kilifi_11865_75_RSVA_2004	2004	Kenya	GA5	5
KU316096	RSVA/Homo sapiens/USA/96E-016-01/1996	1996	USA	GA5	Das, S.R. et al.
KU316104	RSVA/Homo sapiens/USA/91P-516-01/1991	1991	USA	GA5	Das, S.R. et al.
KU316121	RSVA/Homo sapiens/USA/92E-015-01/1992	1992	USA	GA5	Das, S.R. et al.
KU316145	RSVA/Homo sapiens/USA/96E-033-01/1996	1996	USA	GA5	Das, S.R. et al.
KU316176	RSVA/Homo sapiens/USA/90I-184A-01-01/1990	1990	USA	GA5	Das, S.R. et al.
KU316180	RSVA/Homo sapiens/USA/98E-415-01/1998	1998	USA	GA5	Das, S.R. et al.
KU950473	RSVA/Homo sapiens/USA/TH_10346/2012	2012	USA	GA5	Das, S.R. et al.
KU950479	RSVA/Homo sapiens/USA/TH_10180/2012	2012	USA	GA5	Das, S.R. et al.
KU950487	RSVA/Homo sapiens/USA/TH_10334/2012	2012	USA	GA5	Das, S.R. et al.
KU950501	RSVA/Homo sapiens/USA/TH_10212/2013	2013	USA	GA5	Das, S.R. et al.
KU950561	RSVB/Homo sapiens/USA/TH_10667/2006	2006	USA	GA5	Das, S.R. et al.
KU950564	RSVA/Homo sapiens/USA/TH_10234/2012	2012	USA	GA5	Das, S.R. et al.
KU950616	RSVA/Homo sapiens/USA/TH_10226/2012	2012	USA	GA5	Das, S.R. et al.

KX765891	RSVA/Homo sapiens/NZL/LJRSV44/2013	2013	New Zealand	GA5	Shabman, R. et al.
KX765933	RSVA/Homo sapiens/NZL/LJRSV58/2012	2012	New Zealand	GA5	Shabman, R. et al.
KY296713	BJ_08-04	2005	China	GA5	9
KY296716	BJ_09-55	2005	China	GA5	9
KY296767	HuN13-10	2013	China	GA5	9
KY967364	SC2632	2015	USA	GA5	Greninger, A.L. et al.
LC337813	HRSV/Yokohama.JPN/V11519/1993	1993	Japan	GA5	Saikusa, M. et al.
LC337814	HRSV/Yokohama.JPN/V12340/1995	1995	Japan	GA5	Saikusa, M. et al.
LC337815	HRSV/Yokohama.JPN/V12391/1995	1995	Japan	GA5	Saikusa, M. et al.
LC337834	HRSV/Yokohama.JPN/P74/1999	1999	Japan	GA5	Saikusa, M. et al.

Supplementary Table S1 (continued). Strains used in this study.

GenBank accession No.	Strain	Collection year	Country	Genotype	References or authorship
LC337835	HRSV/Yokohama.JPN/P121/1999	1999	Japan	GA5	Saikusa, M. et al.
LC337838	HRSV/Yokohama.JPN/P182/1999	1999	Japan	GA5	Saikusa, M. et al.
LC337845	HRSV/Yokohama.JPN/P208/1999	1999	Japan	GA5	Saikusa, M. et al.
LC337847	HRSV/Yokohama.JPN/P225/2000	2000	Japan	GA5	Saikusa, M. et al.
LC337848	HRSV/Yokohama.JPN/P302/2000	2000	Japan	GA5	Saikusa, M. et al.
LC337849	HRSV/Yokohama.JPN/P337/2000	2000	Japan	GA5	Saikusa, M. et al.
LC337850	HRSV/Yokohama.JPN/P841/2000	2000	Japan	GA5	Saikusa, M. et al.
LC337852	HRSV/Yokohama.JPN/P881/2000	2000	Japan	GA5	Saikusa, M. et al.
LC337855	HRSV/Yokohama.JPN/P909/2000	2000	Japan	GA5	Saikusa, M. et al.
LC337856	HRSV/Yokohama.JPN/P936/2001	2001	Japan	GA5	Saikusa, M. et al.
LC337859	HRSV/Yokohama.JPN/P1255/2001	2001	Japan	GA5	Saikusa, M. et al.
LC337860	HRSV/Yokohama.JPN/P1298/2001	2001	Japan	GA5	Saikusa, M. et al.
LC337864	HRSV/Yokohama.JPN/P1361/2001	2001	Japan	GA5	Saikusa, M. et al.
LC337867	HRSV/Yokohama.JPN/P1371/2001	2001	Japan	GA5	Saikusa, M. et al.
LC337868	HRSV/Yokohama.JPN/P1376/2001	2001	Japan	GA5	Saikusa, M. et al.
LC337871	HRSV/Yokohama.JPN/P1401/2001	2001	Japan	GA5	Saikusa, M. et al.
LC337873	HRSV/Yokohama.JPN/P1407/2001	2001	Japan	GA5	Saikusa, M. et al.
LC337874	HRSV/Yokohama.JPN/P1416/2001	2001	Japan	GA5	Saikusa, M. et al.
LC337876	HRSV/Yokohama.JPN/P1426/2001	2001	Japan	GA5	Saikusa, M. et al.
LC337877	HRSV/Yokohama.JPN/P1427/2001	2001	Japan	GA5	Saikusa, M. et al.
LC337884	HRSV/Yokohama.JPN/P1451/2001	2001	Japan	GA5	Saikusa, M. et al.
LC337885	HRSV/Yokohama.JPN/P1452/2001	2001	Japan	GA5	Saikusa, M. et al.
LC337887	HRSV/Yokohama.JPN/P1891/2002	2002	Japan	GA5	Saikusa, M. et al.
LC337888	HRSV/Yokohama.JPN/P1905/2002	2002	Japan	GA5	Saikusa, M. et al.
LC337890	HRSV/Yokohama.JPN/P1924/2002	2002	Japan	GA5	Saikusa, M. et al.
LC337891	HRSV/Yokohama.JPN/P2037/2003	2003	Japan	GA5	Saikusa, M. et al.
LC337893	HRSV/Yokohama.JPN/P2710/2004	2004	Japan	GA5	Saikusa, M. et al.
LC337903	HRSV/Yokohama.JPN/P2769/2004	2004	Japan	GA5	Saikusa, M. et al.
LC337905	HRSV/Yokohama.JPN/P2785/2004	2004	Japan	GA5	Saikusa, M. et al.
LC337906	HRSV/Yokohama.JPN/P2790/2004	2004	Japan	GA5	Saikusa, M. et al.
LC337907	HRSV/Yokohama.JPN/P2791/2004	2004	Japan	GA5	Saikusa, M. et al.
LC337909	HRSV/Yokohama.JPN/P3151/2005	2005	Japan	GA5	Saikusa, M. et al.
LC365790	HRSV/Yokohama.JPN/P4094/2007	2007	Japan	GA5	Saikusa, M. et al.
MF001038	1D1	2015	USA	GA5	Greninger, A.L. et al.
MF978512	RSVA-CAP-9/BJ/2014	2015	China	GA5	12
MF978625	RSVA-WZ-131/ZJ/2015	2015	China	GA5	12
MG027862	RSV-A/US/BID-V8392/2003	2003	USA	GA5	Newman, R.M. et al.
MG642048	RSVA/Homo sapiens/USA/MCRSV_258/1990	1990	USA	GA5	Shabman, R. et al.
MG642052	RSVA/Homo sapiens/USA/MCRSV_263/1994	1994	USA	GA5	Shabman, R. et al.
MG642055	RSVA/Homo sapiens/USA/MCRSV_259/1990	1990	USA	GA5	Shabman, R. et al.
MG642061	RSVA/Homo sapiens/USA/MCRSV_262/1994	1994	USA	GA5	Shabman, R. et al.
MH760599	A/NSW/WM2955A/10	2010	Australia	GA5	13
KP258723	RSVA/Homo sapiens/USA/86E-007-01/1986	1986	USA	GA6	Das, S.R. et al.
KP258733	RSVA/Homo sapiens/USA/84E-004-01/1984	1984	USA	GA6	Das, S.R. et al.
KU316125	RSVA/Homo sapiens/USA/84I-266A-01-01/1984	1984	USA	GA6	Das, S.R. et al.
KU316138	RSVA/Homo sapiens/USA/87E-083-01/1987	1987	USA	GA6	Das, S.R. et al.
MG642063	RSVA/Homo sapiens/USA/MCRSV_226/1982	1982	USA	GA6	Shabman, R. et al.
MG642070	RSVA/Homo sapiens/USA/MCRSV_247/1986	1986	USA	GA6	Shabman, R. et al.
JF920062	A/WI/629-3248/98	1998	USA	GA7	3

JF920065	A/WI/629-4071/98	1998	USA	GA7	3
JX069800	RSVA/human/USA/A1997-12-35/1997	1997	USA	GA7	Lorenzi, H. et al.
JX069801	RSVA/human/USA/A1998-03-02/1998	1998	USA	GA7	Lorenzi, H. et al.
JX198132	TX-79303	2005	USA	GA7	1
KJ723464	RSVA/Homo sapiens/USA/89I-150A-01/1989	1989	USA	GA7	Das, S.R. et al.
KJ723492	RSVA/Homo sapiens/USA/90I-226A-01/1990	1990	USA	GA7	Das, S.R. et al.
KP258700	RSVA/Homo sapiens/USA/85E-107-01/1985	1985	USA	GA7	Das, S.R. et al.
KP258734	RSVA/Homo sapiens/USA/89E-024-01/1989	1989	USA	GA7	Das, S.R. et al.
KP258737	RSVA/Homo sapiens/USA/84E-016-01/1984	1984	USA	GA7	Das, S.R. et al.
KP258743	RSVA/Homo sapiens/USA/98E-027-01/1998	1998	USA	GA7	Das, S.R. et al.

Supplementary Table S1 (continued). Strains used in this study.

GenBank accession No.	Strain	Collection year	Country	Genotype	References or authorship
KU316092	RSVA/Homo sapiens/USA/91E-610-01/1991	1991	USA	GA7	Das, S.R. et al.
KU316110	RSVA/Homo sapiens/USA/84I-220A-01-01/1984	1984	USA	GA7	Das, S.R. et al.
KU316167	RSVA/Homo sapiens/USA/85I-040A-01-01/1985	1985	USA	GA7	Das, S.R. et al.
LC337825	HRSV/Yokohama.JPN/V14460/1997	1997	Japan	GA7	Saikusa, M. et al.
LC337827	HRSV/Yokohama.JPN/V14761/1997	1997	Japan	GA7	Saikusa, M. et al.
LC337828	HRSV/Yokohama.JPN/V14775/1997	1997	Japan	GA7	Saikusa, M. et al.
LC337831	HRSV/Yokohama.JPN/V14824/1997	1997	Japan	GA7	Saikusa, M. et al.
LC337832	HRSV/Yokohama.JPN/V14827/1997	1997	Japan	GA7	Saikusa, M. et al.
LC337836	HRSV/Yokohama.JPN/P158/1999	1999	Japan	GA7	Saikusa, M. et al.
LC337839	HRSV/Yokohama.JPN/P191/1999	1999	Japan	GA7	Saikusa, M. et al.
LC337841	HRSV/Yokohama.JPN/P200/1999	1999	Japan	GA7	Saikusa, M. et al.
LC337843	HRSV/Yokohama.JPN/P206/1999	1999	Japan	GA7	Saikusa, M. et al.
LC337844	HRSV/Yokohama.JPN/P207/1999	1999	Japan	GA7	Saikusa, M. et al.
LC337851	HRSV/Yokohama.JPN/P850/2000	2000	Japan	GA7	Saikusa, M. et al.
LC337853	HRSV/Yokohama.JPN/P889/2000	2000	Japan	GA7	Saikusa, M. et al.
LC337857	HRSV/Yokohama.JPN/P946/2001	2001	Japan	GA7	Saikusa, M. et al.
LC337889	HRSV/Yokohama.JPN/P1919/2002	2002	Japan	GA7	Saikusa, M. et al.
LC337899	HRSV/Yokohama.JPN/P2762/2004	2004	Japan	GA7	Saikusa, M. et al.
LC337900	HRSV/Yokohama.JPN/P2765/2004	2004	Japan	GA7	Saikusa, M. et al.
LC337901	HRSV/Yokohama.JPN/P2766/2004	2004	Japan	GA7	Saikusa, M. et al.
LC337916	HRSV/Yokohama.JPN/P3233/2005	2005	Japan	GA7	Saikusa, M. et al.
MG642030	RSVA/Homo sapiens/USA/MCRSV_256/1988	1988	USA	GA7	Shabman, R. et al.
MG642034	RSVA/Homo sapiens/USA/MCRSV_244/1985	1985	USA	GA7	Shabman, R. et al.
AB848366	BPH-12-069	2012	Philippines	NA1	Okamoto, M. et al.
AB848369	BPH-13-025	2013	Philippines	NA1	Okamoto, M. et al.
AB848370	ONP-12-144	2012	Philippines	NA1	Okamoto, M. et al.
AB848371	ONP-12-175	2012	Philippines	NA1	Okamoto, M. et al.
AB848372	ONP-12-188	2012	Philippines	NA1	Okamoto, M. et al.
AB848373	ONP-13-005	2013	Philippines	NA1	Okamoto, M. et al.
AB848374	ONP-13-008	2013	Philippines	NA1	Okamoto, M. et al.
JF714709	Riyadh 1/2008	2008	Saudi Arabia	NA1	Farrag, M.A. et al.
JF714710	Riyadh 91/2009	2009	Saudi Arabia	NA1	Farrag, M.A. et al.
JF920046	A/WI/629-2/07	2007	USA	NA1	3
JF920047	A/WI/629-23/08	2008	USA	NA1	3
JF920048	A/WI/629-9-2/07	2007	USA	NA1	3
JF920049	A/WI/629-22/07	2007	USA	NA1	3
JF920054	A/WI/629-Q0282/10	2010	USA	NA1	3
JX015479	11-000271	2011	Netherlands	NA1	4
JX015481	09-000457	2009	Netherlands	NA1	4
JX015482	BE06-6650	2006	Belgium	NA1	4
JX015483	08-047045	2008	Netherlands	NA1	4
JX015490	RSV597	2007	Netherlands	NA1	4
JX015493	08-000507	2008	Netherlands	NA1	4
JX015494	08-001411	2008	Netherlands	NA1	4
JX015495	08-042544	2008	Netherlands	NA1	4
JX015496	08-042735	2008	Netherlands	NA1	4
JX015497	08-044640	2008	Netherlands	NA1	4
JX015498	08-046972	2008	Netherlands	NA1	4
JX015499	BE08-5146	2008	Belgium	NA1	4

JX482019	CQ_Jan-2012(8)	2012	China	NA1	7
JX482020	CQ_Jan-2012(4)	2012	China	NA1	7
JX482021	CQ_Jan-2012(19)	2012	China	NA1	7
JX482022	CQ_Jan-2012(18)	2012	China	NA1	7
JX482023	CQ_Jan-2012(15)	2012	China	NA1	7
JX482024	CQ_Jan-2012(14)	2012	China	NA1	7
JX482025	CQ_Jan-2012(13)	2012	China	NA1	7
JX482026	CQ_Jan-2012(12)	2012	China	NA1	7
JX482027	CQ_Jan-2012(11)	2012	China	NA1	7
JX482028	CQ_Jan-2012(10)	2012	China	NA1	7

Supplementary Table S1 (continued). Strains used in this study.

GenBank accession No.	Strain	Collection year	Country	Genotype	References or authorship
JX482029	CQ_Jan-2012(1)	2012	China	NA1	7
JX482030	CQ_Dec-2011(27)	2011	China	NA1	7
JX482031	CQ_Dec-2011(25)	2011	China	NA1	7
JX482032	CQ_Dec-2011(23)	2011	China	NA1	7
JX482034	CQ_Dec-2011(21)	2011	China	NA1	7
JX482035	CQ_Dec-2011(18)	2011	China	NA1	7
JX482036	CQ_Dec-2011(17)	2011	China	NA1	7
JX482037	CQ_Dec-2011(16)	2011	China	NA1	7
JX482038	CQ_Dec-2011(15)	2011	China	NA1	7
JX627336	RSVA/GN435/11	2011	South Korea	NA1	14
JX682715	CQ_Mar-2012/3163	2012	China	NA1	7
JX682717	CQ_Mar-2012/3174	2012	China	NA1	7
JX682718	CQ_Apr-2012/3229	2012	China	NA1	7
JX682719	CQ_Oct-2011/2251	2011	China	NA1	7
JX682720	CQ_Oct-2011/2310	2011	China	NA1	7
JX682721	CQ_Oct-2011/2315	2011	China	NA1	7
JX682722	CQ_Oct-2011/2319	2011	China	NA1	7
JX682723	CQ_Nov-2011/2348	2011	China	NA1	7
JX682724	CQ_Nov-2011/2353	2011	China	NA1	7
JX682726	CQ_Dec-2011/2580	2011	China	NA1	7
JX682727	CQ_Dec-2011/2587	2011	China	NA1	7
JX682733	CQ_Jan-2012/2792	2012	China	NA1	7
JX682734	CQ_Jan-2012/2848	2012	China	NA1	7
JX682735	CQ_Jan-2012/2881	2012	China	NA1	7
JX682736	CQ_Jan-2012/2904	2012	China	NA1	7
JX682737	CQ_Jan-2012/2907	2012	China	NA1	7
JX682738	CQ_Jan-2012/2947	2012	China	NA1	7
JX682739	CQ_Feb-2012/2992	2012	China	NA1	7
JX682740	CQ_Sep-2010/857	2012	China	NA1	7
JX682741	CQ_Oct-2010/996	2010	China	NA1	7
JX682744	CQ_Dec-2010/1146	2010	China	NA1	7
JX682746	CQ_Dec-2010/1178	2010	China	NA1	7
JX682749	CQ_Dec-2010/1200	2010	China	NA1	7
JX682751	CQ_Jan-2011/1231	2011	China	NA1	7
JX682752	CQ_Jan-2011/1275	2011	China	NA1	7
JX682753	CQ_Jan-2011/1282	2011	China	NA1	7
JX682754	CQ_Feb-2011/1366	2011	China	NA1	7
JX682756	CQ_Feb-2011/1373	2011	China	NA1	7
JX682757	CQ_Feb-2011/1399	2011	China	NA1	7
JX682758	CQ_Mar-2011/1434	2011	China	NA1	7
JX682759	CQ_Mar-2011/1437	2011	China	NA1	7
JX682760	CQ_Mar-2011/1442	2011	China	NA1	7
JX682762	CQ_Mar-2011/1455	2011	China	NA1	7
JX682763	CQ_Mar-2011/1465	2011	China	NA1	7
JX682766	CQ_Apr-2011/1555	2011	China	NA1	7
JX682767	CQ_Apr-2011/1642	2011	China	NA1	7
JX682768	CQ_Apr-2011/1643	2011	China	NA1	7
JX682769	CQ_May-2011/1685	2011	China	NA1	7
JX682770	CQ_May-2011/1712	2011	China	NA1	7

JX682772	CQ_May-2011/1748	2011	China	NA1	7
JX682773	CQ_May-2011/1762	2011	China	NA1	7
JX682774	CQ_Jun-2011/1795	2011	China	NA1	7
JX682775	CQ_Jun-2011/1827	2011	China	NA1	7
JX682777	CQ_Jun-2011/1868	2011	China	NA1	7
JX682778	CQ_Jul-2011/1920	2011	China	NA1	7
JX682779	CQ_Jul-2011/1925	2011	China	NA1	7
JX682781	CQ_Jul-2011/1940	2011	China	NA1	7
JX682782	CQ_Sep-2011/2111	2011	China	NA1	7
JX682783	CQ_Sep-2011/2118	2011	China	NA1	7

Supplementary Table S1 (continued). Strains used in this study.

GenBank accession No.	Strain	Collection year	Country	Genotype	References or authorship
JX682784	CQ_Sep-2011/2147	2011	China	NA1	7
JX682785	CQ_Oct-2011/2208	2011	China	NA1	7
JX682789	CQ_Oct-2011/2247	2011	China	NA1	7
JX682790	CQ_Nov-2011/2362	2011	China	NA1	7
JX682791	CQ_Nov-2011/2379	2011	China	NA1	7
JX682792	CQ_Nov-2011/2384	2011	China	NA1	7
JX682795	CQ_Dec-2011/2626	2011	China	NA1	7
JX682796	CQ_Dec-2011/2627	2011	China	NA1	7
JX682797	CQ_Dec-2011/2628	2011	China	NA1	7
KC618408	9393	2007	Italy	NA1	Piralla, A. et al.
KC618409	9395	2007	Italy	NA1	Piralla, A. et al.
KC618410	9830	2009	Italy	NA1	Piralla, A. et al.
KC731482	RSV-A/NIV1114046/11	2011	India	NA1	15
KC731483	RSV-A/NIV1114073/11	2011	India	NA1	15
KC978856	A/Chongqing/13-73/11.or	2011	China	NA1	16
KF530261	RSVA/Homo sapiens/DEU/106/2008	2008	Germany	NA1	Lorenzi, H. et al.
KF530269	RSVA/Hep2_lab/USA/629-Q0030_RSV60/2009	2009	USA	NA1	Lorenzi, H. et al.
KF826817	RSVA/Homo sapiens/MEX/43/2009	2009	Mexico	NA1	Lorenzi, H. et al.
KF826830	RSVA/Homo sapiens/DEU/107/2009	2009	Germany	NA1	Lorenzi, H. et al.
KF826831	RSVA/Homo sapiens/DEU/108/2009	2009	Germany	NA1	Lorenzi, H. et al.
KF826833	RSVA/Homo sapiens/ITA/121/2009	2009	Italy	NA1	Lorenzi, H. et al.
KF826838	RSVA/Homo sapiens/ARG/177/2006	2006	Argentina	NA1	Lorenzi, H. et al.
KF826840	RSVA/Homo sapiens/MEX/29/2007	2007	Mexico	NA1	Lorenzi, H. et al.
KF826849	RSVA/Hep2_lab/USA/629-Q0115_RSV89/2010	2010	USA	NA1	Lorenzi, H. et al.
KF826855	RSVA/Homo sapiens/ITA/123/2009	2009	Italy	NA1	Lorenzi, H. et al.
KF826856	RSVA/Homo sapiens/ITA/125/2009	2009	Italy	NA1	Lorenzi, H. et al.
KJ130649	CQ_Nov-2012/3998	2012	China	NA1	17
KJ130651	CQ_Jan-2013/4219	2013	China	NA1	17
KJ130653	CQ_May-2013/4945	2013	China	NA1	17
KJ627250	RSVA/Homo sapiens/PER/FLI1381/2010	2010	Peru	NA1	Wentworth, D.E. et al.
KJ627253	RSVA/Homo sapiens/PER/FLE3832/2009	2009	Peru	NA1	Wentworth, D.E. et al.
KJ627257	RSVA/Homo sapiens/PER/FLA5809/2009	2009	Peru	NA1	Wentworth, D.E. et al.
KJ627258	RSVA/Homo sapiens/PER/FLE9486/2010	2010	Peru	NA1	Wentworth, D.E. et al.
KJ627259	RSVA/Homo sapiens/PER/FPI01734/2011	2011	Peru	NA1	Wentworth, D.E. et al.
KJ627264	RSVA/Homo sapiens/PER/IPE01270/2012	2012	Peru	NA1	Wentworth, D.E. et al.
KJ627266	RSVA/Homo sapiens/PER/FLA6730/2009	2009	Peru	NA1	Wentworth, D.E. et al.
KJ627271	RSVA/Homo sapiens/PER/FLE0433/2009	2009	Peru	NA1	Wentworth, D.E. et al.
KJ627272	RSVA/Homo sapiens/PER/FLA6795/2009	2009	Peru	NA1	Wentworth, D.E. et al.
KJ627274	RSVA/Homo sapiens/PER/FLA6534/2009	2009	Peru	NA1	Wentworth, D.E. et al.
KJ627275	RSVA/Homo sapiens/PER/FLE9863/2010	2010	Peru	NA1	Wentworth, D.E. et al.
KJ627282	RSVA/Homo sapiens/PER/FLA6623/2009	2009	Peru	NA1	Wentworth, D.E. et al.
KJ627286	RSVA/Homo sapiens/PER/FLA6646/2009	2009	Peru	NA1	Wentworth, D.E. et al.
KJ627288	RSVA/Homo sapiens/PER/FLA7778/2009	2009	Peru	NA1	Wentworth, D.E. et al.
KJ627293	RSVA/Homo sapiens/PER/FLA7768/2009	2009	Peru	NA1	Wentworth, D.E. et al.
KJ627294	RSVA/Homo sapiens/PER/FLE9761/2010	2010	Peru	NA1	Wentworth, D.E. et al.
KJ627296	RSVA/Homo sapiens/PER/FLE9488/2010	2010	Peru	NA1	Wentworth, D.E. et al.
KJ627300	RSVA/Homo sapiens/PER/FLA7447/2009	2009	Peru	NA1	Wentworth, D.E. et al.
KJ627307	RSVA/Homo sapiens/PER/FLE9867/2010	2010	Peru	NA1	Wentworth, D.E. et al.
KJ627308	RSVA/Homo sapiens/PER/FLE8972/2010	2010	Peru	NA1	Wentworth, D.E. et al.

KJ627312	RSVA/Homo sapiens/PER/FLE9996/2010	2010	Peru	NA1	Wentworth, D.E. et al.
KJ627315	RSVA/Homo sapiens/PER/FLA7756/2009	2009	Peru	NA1	Wentworth, D.E. et al.
KJ627316	RSVA/Homo sapiens/PER/FLE9593/2010	2010	Peru	NA1	Wentworth, D.E. et al.
KJ627318	RSVA/Homo sapiens/PER/FLA6745/2009	2009	Peru	NA1	Wentworth, D.E. et al.
KJ627320	RSVA/Homo sapiens/PER/FLE9490/2010	2010	Peru	NA1	Wentworth, D.E. et al.
KJ627322	RSVA/Homo sapiens/PER/FLA6536/2009	2009	Peru	NA1	Wentworth, D.E. et al.
KJ627325	RSVA/Homo sapiens/PER/FLE8626/2010	2010	Peru	NA1	Wentworth, D.E. et al.
KJ627328	RSVA/Homo sapiens/PER/FLI1310/2010	2010	Peru	NA1	Wentworth, D.E. et al.
KJ627333	RSVA/Homo sapiens/PER/FLE8485/2010	2010	Peru	NA1	Wentworth, D.E. et al.
KJ627334	RSVA/Homo sapiens/PER/FLI2436/2010	2010	Peru	NA1	Wentworth, D.E. et al.

Supplementary Table S1 (continued). Strains used in this study.

GenBank accession No.	Strain	Collection year	Country	Genotype	References or authorship
KJ627337	RSVA/Homo sapiens/PER/FLA7776/2009	2009	Peru	NA1	Wentworth, D.E. et al.
KJ627338	RSVA/Homo sapiens/PER/FLA7437/2009	2009	Peru	NA1	Wentworth, D.E. et al.
KJ627344	RSVA/Homo sapiens/PER/FLA6900/2009	2009	Peru	NA1	Wentworth, D.E. et al.
KJ627349	RSVA/Homo sapiens/PER/FLE4190/2009	2009	Peru	NA1	Wentworth, D.E. et al.
KJ627350	RSVA/Homo sapiens/PER/FLA6714/2009	2009	Peru	NA1	Wentworth, D.E. et al.
KJ627351	RSVA/Homo sapiens/PER/FPP00421/2011	2011	Peru	NA1	Wentworth, D.E. et al.
KJ627354	RSVA/Homo sapiens/PER/FLA6683/2009	2009	Peru	NA1	Wentworth, D.E. et al.
KJ627355	RSVA/Homo sapiens/PER/FLE8228/2010	2010	Peru	NA1	Wentworth, D.E. et al.
KJ627360	RSVA/Homo sapiens/PER/FLA7449/2009	2009	Peru	NA1	Wentworth, D.E. et al.
KJ627370	RSVA/Homo sapiens/PER/FPP00538/2011	2011	Peru	NA1	Wentworth, D.E. et al.
KJ627371	RSVA/Homo sapiens/PER/FLE9518/2010	2010	Peru	NA1	Wentworth, D.E. et al.
KJ627372	RSVA/Homo sapiens/PER/FLE8161/2010	2010	Peru	NA1	Wentworth, D.E. et al.
KJ627373	RSVA/Homo sapiens/PER/FLA7446/2009	2009	Peru	NA1	Wentworth, D.E. et al.
KJ627374	RSVA/Homo sapiens/PER/FPP00438/2011	2011	Peru	NA1	Wentworth, D.E. et al.
KJ627737	RSV-A/US/BID-V8725/2013	2013	USA	NA1	Newman, R.M. et al.
KJ672424	RSVA/Homo sapiens/USA/LA2_106/2012	2012	USA	NA1	Das, S. et al.
KJ672427	RSVA/Homo sapiens/USA/LA2_09/2012	2012	USA	NA1	Das, S. et al.
KJ672428	RSVA/Homo sapiens/USA/LA2_55/2013	2013	USA	NA1	Das, S. et al.
KJ672431	RSVA/Homo sapiens/USA/LA2_14/2012	2012	USA	NA1	Das, S. et al.
KJ672432	RSVA/Homo sapiens/USA/LA2_72/2013	2013	USA	NA1	Das, S. et al.
KJ672433	RSVA/Homo sapiens/USA/LA2_95/2013	2013	USA	NA1	Das, S. et al.
KJ672436	RSVA/Homo sapiens/USA/LA2_30/2012	2012	USA	NA1	Das, S. et al.
KJ672440	RSVA/Homo sapiens/USA/LA2_22/2012	2012	USA	NA1	Das, S. et al.
KJ672441	RSVA/Homo sapiens/USA/LA2_87/2013	2013	USA	NA1	Das, S. et al.
KJ672442	RSVA/Homo sapiens/USA/LA2_45/2013	2013	USA	NA1	Das, S. et al.
KJ672443	RSVA/Homo sapiens/USA/LA2_21/2013	2013	USA	NA1	Das, S. et al.
KJ672444	RSVA/Homo sapiens/USA/LA2_11/2012	2012	USA	NA1	Das, S. et al.
KJ672448	RSVA/Homo sapiens/USA/LA2_74/2013	2013	USA	NA1	Das, S. et al.
KJ672449	RSVA/Homo sapiens/USA/LA2_28/2012	2012	USA	NA1	Das, S. et al.
KJ672454	RSVA/Homo sapiens/USA/LA2_73/2013	2013	USA	NA1	Das, S. et al.
KJ672455	RSVA/Homo sapiens/USA/LA2_05/2012	2012	USA	NA1	Das, S. et al.
KJ672458	RSVA/Homo sapiens/USA/LA2_77/2013	2013	USA	NA1	Das, S. et al.
KJ672459	RSVA/Homo sapiens/USA/LA2_60/2013	2013	USA	NA1	Das, S. et al.
KJ672464	RSVA/Homo sapiens/USA/LA2_04/2012	2012	USA	NA1	Das, S. et al.
KJ672465	RSVA/Homo sapiens/USA/LA2_13/2012	2012	USA	NA1	Das, S. et al.
KJ672469	RSVA/Homo sapiens/USA/LA2_44/2013	2013	USA	NA1	Das, S. et al.
KJ672471	RSVA/Homo sapiens/USA/LA2_85/2013	2013	USA	NA1	Das, S. et al.
KJ672472	RSVA/Homo sapiens/USA/LA2_15/2012	2012	USA	NA1	Das, S. et al.
KJ672475	RSVA/Homo sapiens/USA/LA2_17/2013	2013	USA	NA1	Das, S. et al.
KJ672478	RSVA/Homo sapiens/USA/LA2_29/2013	2013	USA	NA1	Das, S. et al.
KJ672480	RSVA/Homo sapiens/USA/LA2_62/2013	2013	USA	NA1	Das, S. et al.
KJ672482	RSVA/Homo sapiens/USA/LA2_41/2012	2012	USA	NA1	Das, S. et al.
KJ939936	VN-120-7/09	2009	Viet Nam	NA1	11
KJ939939	VN-168-10/09	2009	Viet Nam	NA1	11
KJ939940	VN-217-12/09	2009	Viet Nam	NA1	11
KJ939942	VN-356-6/10	2010	Viet Nam	NA1	11
KJ939945	VN-372-6/10	2010	Viet Nam	NA1	11
KJ939946	VN-378-7/10	2010	Viet Nam	NA1	11
KJ939949	VN-525-3/10	2010	Viet Nam	NA1	11

KJ939950	VN-587-7/10	2010	Viet Nam	NA1	11
KJ939951	VN-733-10/09	2009	Viet Nam	NA1	11
KJ939961	VN-794-7/10	2010	Viet Nam	NA1	11
KJ939963	VN-808-8/10	2010	Viet Nam	NA1	11
KJ939964	VN-816-8/10	2010	Viet Nam	NA1	11
KJ939965	VN-820-8/10	2010	Viet Nam	NA1	11
KJ939966	VN-824-9/10	2010	Viet Nam	NA1	11
KJ939967	VN-832-9/10	2010	Viet Nam	NA1	11
KJ939968	VN-833-9/10	2010	Viet Nam	NA1	11
KJ939969	VN-834-9/10	2010	Viet Nam	NA1	11

Supplementary Table S1 (continued). Strains used in this study.

GenBank accession No.	Strain	Collection year	Country	Genotype	References or authorship
KJ939970	VN-844-11/10	2010	Viet Nam	NA1	11
KJ939971	VN-848-12/10	2010	Viet Nam	NA1	11
KM042382	RSVA/Homo sapiens/USA/LA2_47/2013	2013	USA	NA1	Das, S. et al.
KM042384	RSVA/Homo sapiens/USA/LA2_78/2013	2013	USA	NA1	Das, S. et al.
KM042385	RSVA/Homo sapiens/USA/LA2_39/2013	2013	USA	NA1	Das, S. et al.
KM042386	RSVA/Homo sapiens/USA/LA2_49/2013	2013	USA	NA1	Das, S. et al.
KM042388	RSVA/Homo sapiens/USA/LA2_98/2013	2013	USA	NA1	Das, S. et al.
KM042389	RSVA/Homo sapiens/USA/LA2_10/2012	2012	USA	NA1	Das, S. et al.
KM042390	RSVA/Homo sapiens/USA/LA2_94/2013	2013	USA	NA1	Das, S. et al.
KM042391	RSVA/Homo sapiens/USA/LA2_83/2013	2013	USA	NA1	Das, S. et al.
KM042392	RSVA/Homo sapiens/USA/LA2_56/2013	2013	USA	NA1	Das, S. et al.
KM517572	A/GZ/11-224	2011	China	NA1	Xie, J.H. et al.
KM578843	A/GZ/12-110	2012	China	NA1	Xie, J.H. et al.
KP119748	HRSV-A-GZ08-19	2012	Hong Kong	NA1	8
KP317920	Kilifi_11862_28_RSVA_2010	2011	Kenya	NA1	5
KP317921	Kilifi_11862_33_RSVA_2010	2010	Kenya	NA1	5
KP317926	Kilifi_10891_60_RSVA_2007	2007	Kenya	NA1	5
KP317935	Kilifi_10899_41_RSVA_2010	2010	Kenya	NA1	5
KP317951	Kilifi_11864_53_RSVA_2011	2011	Kenya	NA1	5
KP317953	Kilifi_11862_29_RSVA_2011	2012	Kenya	NA1	5
KP317955	Kilifi_10028_12_RSVA_2003	2003	Kenya	NA1	5
KP663728	HRSV-A/IC688/12	2012	South Korea	NA1	18
KT285064	B614122905	2014	China	NA1	19
KU839630	RSVA/Homo sapiens/USA/TH-28/2014	2014	USA	NA1	Das, S.R. et al.
KU839637	RSVA/Homo sapiens/USA/TH-24/2014	2014	USA	NA1	Das, S.R. et al.
KU950460	RSVA/Homo sapiens/USA/TH_10473/2013	2013	USA	NA1	Das, S.R. et al.
KU950464	RSVA/Homo sapiens/USA/TH_10654/2014	2014	USA	NA1	Das, S.R. et al.
KU950480	RSVA/Homo sapiens/USA/TH_10272/2012	2012	USA	NA1	Das, S.R. et al.
KU950483	RSVA/Homo sapiens/USA/TH_10534/2013	2013	USA	NA1	Das, S.R. et al.
KU950485	RSVA/Homo sapiens/USA/TH_10210/2012	2012	USA	NA1	Das, S.R. et al.
KU950486	RSVA/Homo sapiens/USA/TH_10280/2012	2012	USA	NA1	Das, S.R. et al.
KU950491	RSVA/Homo sapiens/USA/TH_10518/2013	2013	USA	NA1	Das, S.R. et al.
KU950492	RSVA/Homo sapiens/USA/TH_10166/2012	2012	USA	NA1	Das, S.R. et al.
KU950505	RSVA/Homo sapiens/USA/TH_10651/2013	2013	USA	NA1	Das, S.R. et al.
KU950511	RSVA/Homo sapiens/USA/TH_10218/2012	2012	USA	NA1	Das, S.R. et al.
KU950513	RSVA/Homo sapiens/USA/TH_10173/2012	2012	USA	NA1	Das, S.R. et al.
KU950519	RSVA/Homo sapiens/USA/TH_10629/2013	2013	USA	NA1	Das, S.R. et al.
KU950521	RSVA/Homo sapiens/USA/TH_10149/2012	2012	USA	NA1	Das, S.R. et al.
KU950523	RSVA/Homo sapiens/USA/TH_10656/2014	2014	USA	NA1	Das, S.R. et al.
KU950524	RSVA/Homo sapiens/USA/TH_10094/2012	2012	USA	NA1	Das, S.R. et al.
KU950529	RSVA/Homo sapiens/USA/TH_10116/2012	2012	USA	NA1	Das, S.R. et al.
KU950531	RSVA/Homo sapiens/USA/TH_10523/2013	2013	USA	NA1	Das, S.R. et al.
KU950541	RSVA/Homo sapiens/USA/TH_10318/2013	2013	USA	NA1	Das, S.R. et al.
KU950549	RSVA/Homo sapiens/USA/TH_10630/2013	2013	USA	NA1	Das, S.R. et al.
KU950550	RSVA/Homo sapiens/USA/TH_10287/2012	2012	USA	NA1	Das, S.R. et al.
KU950556	RSVA/Homo sapiens/USA/TH_10305/2012	2012	USA	NA1	Das, S.R. et al.
KU950567	RSVA/Homo sapiens/USA/TH_10324/2012	2012	USA	NA1	Das, S.R. et al.
KU950581	RSVA/Homo sapiens/USA/TH_10159/2012	2012	USA	NA1	Das, S.R. et al.
KU950585	RSVB/Homo sapiens/USA/TH_10613/2013	2013	USA	NA1	Das, S.R. et al.

KU950590	RSVA/Homo sapiens/USA/TH_10387/2013	2013	USA	NA1	Das, S.R. et al.
KU950594	RSVA/Homo sapiens/USA/TH_10538/2013	2013	USA	NA1	Das, S.R. et al.
KU950598	RSVA/Homo sapiens/USA/TH_10618/2013	2013	USA	NA1	Das, S.R. et al.
KU950608	RSVA/Homo sapiens/USA/TH_10508/2013	2013	USA	NA1	Das, S.R. et al.
KU950610	RSVA/Homo sapiens/USA/TH_10260/2013	2013	USA	NA1	Das, S.R. et al.
KU950612	RSVA/Homo sapiens/USA/TH_10281/2012	2012	USA	NA1	Das, S.R. et al.
KU950617	RSVA/Homo sapiens/USA/TH_10229/2012	2012	USA	NA1	Das, S.R. et al.
KU950623	RSVA/Homo sapiens/USA/TH_10606/2013	2013	USA	NA1	Das, S.R. et al.
KU950624	RSVA/Homo sapiens/USA/TH_10337/2013	2013	USA	NA1	Das, S.R. et al.
KU950626	RSVA/Homo sapiens/USA/TH_10207/2013	2013	USA	NA1	Das, S.R. et al.

Supplementary Table S1 (continued). Strains used in this study.

GenBank accession No.	Strain	Collection year	Country	Genotype	References or authorship
KU950627	RSVA/Homo sapiens/USA/TH_10569/2013	2013	USA	NA1	Das, S.R. et al.
KU950628	RSVA/Homo sapiens/USA/TH_10364/2013	2013	USA	NA1	Das, S.R. et al.
KU950629	RSVA/Homo sapiens/USA/TH_10500/2013	2013	USA	NA1	Das, S.R. et al.
KU950631	RSVA/Homo sapiens/USA/TH_10093/2012	2012	USA	NA1	Das, S.R. et al.
KU950638	RSVA/Homo sapiens/USA/TH_10552/2013	2013	USA	NA1	Das, S.R. et al.
KU950642	RSVA/Homo sapiens/USA/TH_10128/2012	2012	USA	NA1	Das, S.R. et al.
KU950643	RSVA/Homo sapiens/USA/TH_10268/2012	2012	USA	NA1	Das, S.R. et al.
KU950649	RSVA/Homo sapiens/USA/TH_11186/2012	2012	USA	NA1	Das, S.R. et al.
KU950652	RSVA/Homo sapiens/USA/TH_10144/2012	2012	USA	NA1	Das, S.R. et al.
KU950655	RSVA/Homo sapiens/USA/TH_10293/2012	2012	USA	NA1	Das, S.R. et al.
KU950661	RSVA/Homo sapiens/USA/TH_10183/2012	2012	USA	NA1	Das, S.R. et al.
KU950666	RSVA/Homo sapiens/USA/TH_10657/2013	2013	USA	NA1	Das, S.R. et al.
KU950667	RSVA/Homo sapiens/USA/TH_10104/2012	2012	USA	NA1	Das, S.R. et al.
KU950670	RSVA/Homo sapiens/USA/TH_10220/2012	2012	USA	NA1	Das, S.R. et al.
KU950673	RSVA/Homo sapiens/USA/TH_10236/2012	2012	USA	NA1	Das, S.R. et al.
KU950677	RSVA/Homo sapiens/USA/TH_10148/2012	2012	USA	NA1	Das, S.R. et al.
KU950685	RSVA/Homo sapiens/USA/TH_10509/2013	2013	USA	NA1	Das, S.R. et al.
KU950686	RSVA/Homo sapiens/USA/TH_10506/2014	2014	USA	NA1	Das, S.R. et al.
KU950694	RSVA/Homo sapiens/USA/TH_10233/2012	2012	USA	NA1	Das, S.R. et al.
KU950696	RSVA/Homo sapiens/USA/TH_10283/2012	2012	USA	NA1	Das, S.R. et al.
KX510137	Kilifi_10590_33_RSVA_2010	2010	Kenya	NA1	Agoti, C.N. et al.
KX510141	Kilifi_10028_7_RSVA_2010	2010	Kenya	NA1	Agoti, C.N. et al.
KX510142	Kilifi_10010_53_RSVA_2010	2010	Kenya	NA1	Agoti, C.N. et al.
KX510157	Kilifi_10028_6_RSVA_2010	2010	Kenya	NA1	Agoti, C.N. et al.
KX510159	Kilifi_10025_30_RSVA_2010	2010	Kenya	NA1	Agoti, C.N. et al.
KX510189	Kilifi_9466_21_RSVA_2010	2010	Kenya	NA1	Agoti, C.N. et al.
KX510223	Kilifi_10028_15_RSVA_2010	2010	Kenya	NA1	Agoti, C.N. et al.
KX510229	Kilifi_10025_25_RSVA_2010	2010	Kenya	NA1	Agoti, C.N. et al.
KX510237	Kilifi_9696_36_RSVA_2010	2010	Kenya	NA1	Agoti, C.N. et al.
KX510243	Kilifi_11862_27_RSVA_2010	2010	Kenya	NA1	Agoti, C.N. et al.
KX510247	Kilifi_10028_8_RSVA_2010	2010	Kenya	NA1	Agoti, C.N. et al.
KX510253	Kilifi_10014_42_RSVA_2010	2010	Kenya	NA1	Agoti, C.N. et al.
KX510261	Kilifi_10014_40_RSVA_2010	2010	Kenya	NA1	Agoti, C.N. et al.
KX655616	RSVA/Homo sapiens/JOR/A0444/2012	2012	Jordan	NA1	Shabman, R. et al.
KX655623	RSVA/Homo sapiens/JOR/B1148/2011	2011	Jordan	NA1	Shabman, R. et al.
KX655626	RSVA/Homo sapiens/JOR/D3721/2013	2013	Jordan	NA1	Shabman, R. et al.
KX655630	RSVA/Homo sapiens/JOR/B1429/2012	2012	Jordan	NA1	Shabman, R. et al.
KX655631	RSVA/Homo sapiens/JOR/C2186/2011	2011	Jordan	NA1	Shabman, R. et al.
KX655632	RSVA/Homo sapiens/JOR/B1543/2012	2012	Jordan	NA1	Shabman, R. et al.
KX655636	RSVA/Homo sapiens/JOR/B1191/2011	2011	Jordan	NA1	Shabman, R. et al.
KX655637	RSVA/Homo sapiens/JOR/C2109/2011	2011	Jordan	NA1	Shabman, R. et al.
KX655638	RSVA/Homo sapiens/JOR/A0398/2012	2012	Jordan	NA1	Shabman, R. et al.
KX655639	RSVA/Homo sapiens/JOR/A0125/2011	2011	Jordan	NA1	Shabman, R. et al.
KX655640	RSVA/Homo sapiens/JOR/D3681/2013	2013	Jordan	NA1	Shabman, R. et al.
KX655643	RSVA/Homo sapiens/JOR/D3156/2011	2011	Jordan	NA1	Shabman, R. et al.
KX655644	RSVA/Homo sapiens/JOR/A0614/2012	2012	Jordan	NA1	Shabman, R. et al.
KX655647	RSVA/Homo sapiens/JOR/A0400/2012	2012	Jordan	NA1	Shabman, R. et al.
KX655650	RSVA/Homo sapiens/JOR/C2147/2011	2011	Jordan	NA1	Shabman, R. et al.
KX655656	RSVA/Homo sapiens/JOR/D3007/2010	2010	Jordan	NA1	Shabman, R. et al.

KX655657	RSVA/Homo sapiens/JOR/D3040/2010	2010	Jordan	NA1	Shabman, R. et al.
KX655658	RSVA/Homo sapiens/JOR/C2659/2012	2012	Jordan	NA1	Shabman, R. et al.
KX655662	RSVA/Homo sapiens/JOR/D3317/2011	2011	Jordan	NA1	Shabman, R. et al.
KX655663	RSVA/Homo sapiens/JOR/D3416/2012	2012	Jordan	NA1	Shabman, R. et al.
KX655665	RSVA/Homo sapiens/JOR/C2769/2013	2013	Jordan	NA1	Shabman, R. et al.
KX655666	RSVA/Homo sapiens/JOR/D3141/2011	2011	Jordan	NA1	Shabman, R. et al.
KX655670	RSVA/Homo sapiens/JOR/B1464/2012	2012	Jordan	NA1	Shabman, R. et al.
KX655672	RSVA/Homo sapiens/JOR/C2356/2012	2012	Jordan	NA1	Shabman, R. et al.
KX655675	RSVA/Homo sapiens/JOR/D3431/2012	2012	Jordan	NA1	Shabman, R. et al.
KX655676	RSVA/Homo sapiens/JOR/B1432/2012	2012	Jordan	NA1	Shabman, R. et al.

Supplementary Table S1 (continued). Strains used in this study.

GenBank accession No.	Strain	Collection year	Country	Genotype	References or authorship
KX655677	RSVA/Homo sapiens/JOR/B1391/2012	2012	Jordan	NA1	Shabman, R. et al.
KX655678	RSVA/Homo sapiens/JOR/A0108/2011	2011	Jordan	NA1	Shabman, R. et al.
KX655679	RSVA/Homo sapiens/JOR/B1035/2010	2010	Jordan	NA1	Shabman, R. et al.
KX655682	RSVA/Homo sapiens/JOR/B1515/2012	2012	Jordan	NA1	Shabman, R. et al.
KX655689	RSVA/Homo sapiens/JOR/B1454/2012	2012	Jordan	NA1	Shabman, R. et al.
KX655691	RSVA/Homo sapiens/JOR/D3371/2012	2012	Jordan	NA1	Shabman, R. et al.
KX655692	RSVA/Homo sapiens/JOR/C2196/2011	2011	Jordan	NA1	Shabman, R. et al.
KX655694	RSVA/Homo sapiens/JOR/A0215/2011	2011	Jordan	NA1	Shabman, R. et al.
KX655696	RSVA/Homo sapiens/JOR/C2121/2011	2011	Jordan	NA1	Shabman, R. et al.
KX655697	RSVA/Homo sapiens/JOR/A0417/2012	2012	Jordan	NA1	Shabman, R. et al.
KX655699	RSVA/Homo sapiens/JOR/D3332/2012	2012	Jordan	NA1	Shabman, R. et al.
KX765894	RSVA/Homo sapiens/NZL/LJRSV84/2011	2011	New Zealand	NA1	Shabman, R. et al.
KX765896	RSVA/Homo sapiens/NZL/LJRSV81/2011	2011	New Zealand	NA1	Shabman, R. et al.
KX765898	RSVA/Homo sapiens/NZL/LJRSV12/2015	2015	New Zealand	NA1	Shabman, R. et al.
KX765902	RSVA/Homo sapiens/NZL/LJRSV33/2014	2014	New Zealand	NA1	Shabman, R. et al.
KX765910	RSVA/Homo sapiens/NZL/LJRSV69/2011	2011	New Zealand	NA1	Shabman, R. et al.
KX765911	RSVA/Homo sapiens/NZL/LJRSV75/2011	2011	New Zealand	NA1	Shabman, R. et al.
KX765916	RSVA/Homo sapiens/NZL/LJRSV02/2015	2015	New Zealand	NA1	Shabman, R. et al.
KX765917	RSVA/Homo sapiens/NZL/LJRSV28/2014	2014	New Zealand	NA1	Shabman, R. et al.
KX765918	RSVA/Homo sapiens/NZL/LJRSV77/2011	2011	New Zealand	NA1	Shabman, R. et al.
KX765919	RSVA/Homo sapiens/NZL/LJRSV37/2013	2013	New Zealand	NA1	Shabman, R. et al.
KX765924	RSVA/Homo sapiens/NZL/LJRSV99/2010	2010	New Zealand	NA1	Shabman, R. et al.
KX765925	RSVA/Homo sapiens/NZL/LJRSV13/2015	2015	New Zealand	NA1	Shabman, R. et al.
KX765926	RSVA/Homo sapiens/NZL/LJRSV59/2012	2012	New Zealand	NA1	Shabman, R. et al.
KX765928	RSVA/Homo sapiens/NZL/LJRSV83/2011	2011	New Zealand	NA1	Shabman, R. et al.
KX765931	RSVA/Homo sapiens/NZL/LJRSV70/2011	2011	New Zealand	NA1	Shabman, R. et al.
KX765932	RSVA/Homo sapiens/NZL/LJRSV06/2015	2015	New Zealand	NA1	Shabman, R. et al.
KX765934	RSVA/Homo sapiens/NZL/LJRSV65/2012	2012	New Zealand	NA1	Shabman, R. et al.
KX765938	RSVA/Homo sapiens/NZL/LJRSV74/2011	2011	New Zealand	NA1	Shabman, R. et al.
KX765941	RSVA/Homo sapiens/NZL/LJRSV04/2015	2015	New Zealand	NA1	Shabman, R. et al.
KX765942	RSVA/Homo sapiens/NZL/LJRSV80/2011	2011	New Zealand	NA1	Shabman, R. et al.
KX765944	RSVA/Homo sapiens/NZL/LJRSV08/2015	2015	New Zealand	NA1	Shabman, R. et al.
KX765946	RSVA/Homo sapiens/NZL/LJRSV57/2012	2012	New Zealand	NA1	Shabman, R. et al.
KX765955	RSVA/Homo sapiens/NZL/LJRSV09/2015	2015	New Zealand	NA1	Shabman, R. et al.
KX765956	RSVA/Homo sapiens/NZL/LJRSV14/2015	2015	New Zealand	NA1	Shabman, R. et al.
KX765958	RSVA/Homo sapiens/NZL/LJRSV68/2012	2012	New Zealand	NA1	Shabman, R. et al.
KX765960	RSVA/Homo sapiens/NZL/LJRSV76/2011	2011	New Zealand	NA1	Shabman, R. et al.
KX765967	RSVA/Homo sapiens/NZL/LJRSV64/2012	2012	New Zealand	NA1	Shabman, R. et al.
KX765969	RSVA/Homo sapiens/NZL/LJRSV61/2012	2012	New Zealand	NA1	Shabman, R. et al.
KX765970	RSVA/Homo sapiens/NZL/LJRSV31/2014	2014	New Zealand	NA1	Shabman, R. et al.
KX765977	RSVA/Homo sapiens/NZL/LJRSV18/2014	2014	New Zealand	NA1	Shabman, R. et al.
KX858757	13-005275	2013	Netherlands	NA1	Coenjaerts, F.E.J.
KX894805	RSVA/Homo sapiens/USA/TH_10293P/2013	2013	USA	NA1	Shabman, R. et al.
KX894807	RSVA/Homo sapiens/USA/TH_10280P/2013	2013	USA	NA1	Shabman, R. et al.
KY296707	CC12-10	2012	China	NA1	9
KY296714	BJ_08-14	2005	China	NA1	9
KY296717	BJ_09-58	2005	China	NA1	9
KY296719	BJ_10-28	2005	China	NA1	9
KY296720	BJ_11-01	2011	China	NA1	9

KY296721	CC09-01	2009	China	NA1	9
KY296722	CC12-09	2012	China	NA1	9
KY296723	CC12-11	2012	China	NA1	9
KY296724	CC12-14	2012	China	NA1	9
KY296725	CC12-16	2012	China	NA1	9
KY296726	CC13-01	2013	China	NA1	9
KY296727	CC13-12	2013	China	NA1	9
KY296729	CC13-21	2013	China	NA1	9
KY296730	CC14-10	2014	China	NA1	9
KY296732	CC14-52	2014	China	NA1	9

Supplementary Table S1 (continued). Strains used in this study.

GenBank accession No.	Strain	Collection year	Country	Genotype	References or authorship
KY296733	CC14-76	2014	China	NA1	9
KY296734	GZ10-01	2005	China	NA1	9
KY296735	GZ10-06	2005	China	NA1	9
KY296736	GZ11-01	2011	China	NA1	9
KY296737	GZ11-04	2011	China	NA1	9
KY296740	GZ11-11	2005	China	NA1	9
KY296741	GZ11-12	2005	China	NA1	9
KY296742	GZ11-13	2005	China	NA1	9
KY296743	GZ11-14	2005	China	NA1	9
KY296744	GZ11-15	2005	China	NA1	9
KY296745	GZ11-16	2005	China	NA1	9
KY296746	GZ11-17	2005	China	NA1	9
KY296749	GZ11-02	2011	China	NA1	9
KY296750	GZ11-20	2005	China	NA1	9
KY296752	GZ11-22	2005	China	NA1	9
KY296753	GZ11-24	2005	China	NA1	9
KY296754	GZ11-25	2005	China	NA1	9
KY296755	GZ11-26	2005	China	NA1	9
KY296756	GZ11-27	2005	China	NA1	9
KY296758	GZ11-05	2011	China	NA1	9
KY296759	GZ11-06	2011	China	NA1	9
KY296760	GZ11-07	2011	China	NA1	9
KY296761	GZ11-08	2011	China	NA1	9
KY296762	GZ12-01	2012	China	NA1	9
KY296763	HeB13-02	2013	China	NA1	9
KY296764	HeB14-01	2014	China	NA1	9
KY296765	HuN12-04	2012	China	NA1	9
KY296766	HuN12-20	2012	China	NA1	9
KY296768	HuN14-02	2005	China	NA1	9
KY296770	HuN14-04	2005	China	NA1	9
KY296771	SaX10-45	2010	China	NA1	9
KY296772	SaX10-46	2010	China	NA1	9
KY296774	SaX10-64	2010	China	NA1	9
KY296775	SaX10-68	2010	China	NA1	9
KY296776	SaX11-01	2011	China	NA1	9
KY296777	SaX11-15	2011	China	NA1	9
KY296778	SaX11-16	2011	China	NA1	9
KY296779	SaX13-43	2013	China	NA1	9
KY296781	GS10-03	2010	China	NA1	9
KY296782	GS10-04	2010	China	NA1	9
KY296786	GS11-08	2011	China	NA1	9
KY296787	GS11-11	2011	China	NA1	9
KY296788	GS11-13	2011	China	NA1	9
KY296790	GS11-20	2011	China	NA1	9
KY296793	HuN11-01	2014	China	NA1	9
KY296794	SH10-10	2010	China	NA1	9
KY296795	SH10-15	2010	China	NA1	9
KY296796	SH10-16	2010	China	NA1	9
KY296797	SH10-17	2010	China	NA1	9

KY460517	TW-00026-2010	2010	Taiwan	NA1	20
KY654506	TTa-12-054	2012	Philippines	NA1	21
KY654508	TEv-12-111	2012	Philippines	NA1	21
KY654509	TEv-12-137	2012	Philippines	NA1	21
KY654510	TOp-12-061	2012	Philippines	NA1	21
KY654511	TBp-13-167	2013	Philippines	NA1	21
KY654514	TEv-13-132	2013	Philippines	NA1	21
KY654516	TOp-13-186	2013	Philippines	NA1	21
KY654517	TOp-13-203	2013	Philippines	NA1	21
KY782635	LZ01/09	2009	China	NA1	Zhu, C. et al.

Supplementary Table S1 (continued). Strains used in this study.

GenBank accession No.	Strain	Collection year	Country	Genotype	References or authorship
KY883566	HRSV/A/BuenosAires/ARG/001/2015	2015	Argentina	NA1	Goya, S. et al.
KY883572	HRSV/A/BuenosAires/ARG/003/2015	2015	Argentina	NA1	Goya, S. et al.
KY967362	SC2892	2015	USA	NA1	Greninger, A.L. et al.
KY967363	SC2745	2015	USA	NA1	Greninger, A.L. et al.
KY982517	RSVA/Homo sapiens/USA/LA2_03/2013	2013	USA	NA1	Shrivastava, S. et al.
LC337896	HRSV/Yokohama.JPN/P2733/2004	2004	Japan	NA1	Saikusa, M. et al.
LC337898	HRSV/Yokohama.JPN/P2747/2004	2004	Japan	NA1	Saikusa, M. et al.
LC337908	HRSV/Yokohama.JPN/P2820/2005	2005	Japan	NA1	Saikusa, M. et al.
LC337910	HRSV/Yokohama.JPN/P3180/2005	2005	Japan	NA1	Saikusa, M. et al.
LC337911	HRSV/Yokohama.JPN/P3189/2005	2005	Japan	NA1	Saikusa, M. et al.
LC337912	HRSV/Yokohama.JPN/P3194/2005	2005	Japan	NA1	Saikusa, M. et al.
LC337913	HRSV/Yokohama.JPN/P3196/2005	2005	Japan	NA1	Saikusa, M. et al.
LC337915	HRSV/Yokohama.JPN/P3227/2005	2005	Japan	NA1	Saikusa, M. et al.
LC365793	HRSV/Yokohama.JPN/P4258/2008	2008	Japan	NA1	Saikusa, M. et al.
LC365794	HRSV/Yokohama.JPN/P4352/2008	2008	Japan	NA1	Saikusa, M. et al.
LC365796	HRSV/Yokohama.JPN/P4396/2008	2008	Japan	NA1	Saikusa, M. et al.
LC365800	HRSV/Yokohama.JPN/P4425/2008	2008	Japan	NA1	Saikusa, M. et al.
LC365802	HRSV/Yokohama.JPN/P4431/2008	2008	Japan	NA1	Saikusa, M. et al.
LC365804	HRSV/Yokohama.JPN/P4434/2008	2008	Japan	NA1	Saikusa, M. et al.
LC365806	HRSV/Yokohama.JPN/P4438/2008	2008	Japan	NA1	Saikusa, M. et al.
LC365807	HRSV/Yokohama.JPN/P4440/2008	2008	Japan	NA1	Saikusa, M. et al.
LC365808	HRSV/Yokohama.JPN/P4443/2008	2008	Japan	NA1	Saikusa, M. et al.
LC365809	HRSV/Yokohama.JPN/P4449/2008	2008	Japan	NA1	Saikusa, M. et al.
LC365810	HRSV/Yokohama.JPN/P4450/2008	2008	Japan	NA1	Saikusa, M. et al.
LC365811	HRSV/Yokohama.JPN/P4456/2008	2008	Japan	NA1	Saikusa, M. et al.
LC365812	HRSV/Yokohama.JPN/P4459/2008	2008	Japan	NA1	Saikusa, M. et al.
LC365813	HRSV/Yokohama.JPN/P4470/2008	2008	Japan	NA1	Saikusa, M. et al.
LC365814	HRSV/Yokohama.JPN/P4486/2008	2008	Japan	NA1	Saikusa, M. et al.
LC365817	HRSV/Yokohama.JPN/P4497/2008	2008	Japan	NA1	Saikusa, M. et al.
LC365818	HRSV/Yokohama.JPN/P4499/2008	2008	Japan	NA1	Saikusa, M. et al.
LC365819	HRSV/Yokohama.JPN/P5050/2009	2009	Japan	NA1	Saikusa, M. et al.
LC365821	HRSV/Yokohama.JPN/P5077/2010	2010	Japan	NA1	Saikusa, M. et al.
LC365822	HRSV/Yokohama.JPN/P5082/2010	2010	Japan	NA1	Saikusa, M. et al.
LC365823	HRSV/Yokohama.JPN/P5114/2010	2010	Japan	NA1	Saikusa, M. et al.
LC365824	HRSV/Yokohama.JPN/P5123/2010	2010	Japan	NA1	Saikusa, M. et al.
LC365825	HRSV/Yokohama.JPN/P5127/2010	2010	Japan	NA1	Saikusa, M. et al.
LC365828	HRSV/Yokohama.JPN/P5142/2010	2010	Japan	NA1	Saikusa, M. et al.
LC365830	HRSV/Yokohama.JPN/P5166/2010	2010	Japan	NA1	Saikusa, M. et al.
LC365831	HRSV/Yokohama.JPN/P5167/2010	2010	Japan	NA1	Saikusa, M. et al.
LC365832	HRSV/Yokohama.JPN/P5172/2010	2010	Japan	NA1	Saikusa, M. et al.
LC365833	HRSV/Yokohama.JPN/P5173/2010	2010	Japan	NA1	Saikusa, M. et al.
LC365834	HRSV/Yokohama.JPN/P5184/2010	2010	Japan	NA1	Saikusa, M. et al.
LC365835	HRSV/Yokohama.JPN/P5187/2010	2010	Japan	NA1	Saikusa, M. et al.
LC365836	HRSV/Yokohama.JPN/P5188/2010	2010	Japan	NA1	Saikusa, M. et al.
LC365837	HRSV/Yokohama.JPN/P5193/2010	2010	Japan	NA1	Saikusa, M. et al.
LC365838	HRSV/Yokohama.JPN/P5201/2010	2010	Japan	NA1	Saikusa, M. et al.
LC365839	HRSV/Yokohama.JPN/P5202/2010	2010	Japan	NA1	Saikusa, M. et al.
LC365840	HRSV/Yokohama.JPN/P5403/2010	2010	Japan	NA1	Saikusa, M. et al.
LC365842	HRSV/Yokohama.JPN/P5484/2010	2010	Japan	NA1	Saikusa, M. et al.

LC365843	HRSV/Yokohama.JPN/P5485/2010	2010	Japan	NA1	Saikusa, M. et al.
LC365844	HRSV/Yokohama.JPN/P5486/2010	2010	Japan	NA1	Saikusa, M. et al.
LC365848	HRSV/Yokohama.JPN/P5513/2010	2010	Japan	NA1	Saikusa, M. et al.
LC365849	HRSV/Yokohama.JPN/P5517/2010	2010	Japan	NA1	Saikusa, M. et al.
LC365850	HRSV/Yokohama.JPN/P5531/2010	2010	Japan	NA1	Saikusa, M. et al.
LC365851	HRSV/Yokohama.JPN/P5567/2010	2010	Japan	NA1	Saikusa, M. et al.
LC365852	HRSV/Yokohama.JPN/P5583/2010	2010	Japan	NA1	Saikusa, M. et al.
LC365853	HRSV/Yokohama.JPN/P5587/2010	2010	Japan	NA1	Saikusa, M. et al.
LC365854	HRSV/Yokohama.JPN/P5591/2010	2010	Japan	NA1	Saikusa, M. et al.
LC365855	HRSV/Yokohama.JPN/P5594/2010	2010	Japan	NA1	Saikusa, M. et al.

Supplementary Table S1 (continued). Strains used in this study.

GenBank accession No.	Strain	Collection year	Country	Genotype	References or authorship
LC365856	HRSV/Yokohama.JPN/P5596/2010	2010	Japan	NA1	Saikusa, M. et al.
LC365857	HRSV/Yokohama.JPN/P5623/2011	2011	Japan	NA1	Saikusa, M. et al.
LC365858	HRSV/Yokohama.JPN/P5649/2011	2011	Japan	NA1	Saikusa, M. et al.
LC365859	HRSV/Yokohama.JPN/P5718/2011	2011	Japan	NA1	Saikusa, M. et al.
LC365862	HRSV/Yokohama.JPN/HN170/2011	2011	Japan	NA1	Saikusa, M. et al.
LC365863	HRSV/Yokohama.JPN/P5845/2011	2011	Japan	NA1	Saikusa, M. et al.
LC365864	HRSV/Yokohama.JPN/P5920/2011	2011	Japan	NA1	Saikusa, M. et al.
LC365865	HRSV/Yokohama.JPN/P5947/2011	2011	Japan	NA1	Saikusa, M. et al.
LC365866	HRSV/Yokohama.JPN/P5952/2011	2011	Japan	NA1	Saikusa, M. et al.
LC365867	HRSV/Yokohama.JPN/P5957/2011	2011	Japan	NA1	Saikusa, M. et al.
LC365868	HRSV/Yokohama.JPN/P6097/2011	2011	Japan	NA1	Saikusa, M. et al.
LC365869	HRSV/Yokohama.JPN/P6098/2011	2011	Japan	NA1	Saikusa, M. et al.
LC365870	HRSV/Yokohama.JPN/P6106/2011	2011	Japan	NA1	Saikusa, M. et al.
LC365871	HRSV/Yokohama.JPN/P6247/2012	2012	Japan	NA1	Saikusa, M. et al.
LC365874	HRSV/Yokohama.JPN/P6442/2012	2012	Japan	NA1	Saikusa, M. et al.
LC365875	HRSV/Yokohama.JPN/P6467/2012	2012	Japan	NA1	Saikusa, M. et al.
LC365877	HRSV/Yokohama.JPN/P6510/2012	2012	Japan	NA1	Saikusa, M. et al.
LC365878	HRSV/Yokohama.JPN/P6511/2012	2012	Japan	NA1	Saikusa, M. et al.
LC365879	HRSV/Yokohama.JPN/P6512/2012	2012	Japan	NA1	Saikusa, M. et al.
LC365880	HRSV/Yokohama.JPN/P6515/2012	2012	Japan	NA1	Saikusa, M. et al.
LC365881	HRSV/Yokohama.JPN/P6529/2012	2012	Japan	NA1	Saikusa, M. et al.
LC365882	HRSV/Yokohama.JPN/P6533/2012	2012	Japan	NA1	Saikusa, M. et al.
LC365883	HRSV/Yokohama.JPN/P6535/2012	2012	Japan	NA1	Saikusa, M. et al.
LC365884	HRSV/Yokohama.JPN/P6538/2012	2012	Japan	NA1	Saikusa, M. et al.
LC365885	HRSV/Yokohama.JPN/P6539/2012	2012	Japan	NA1	Saikusa, M. et al.
LC365886	HRSV/Yokohama.JPN/P6540/2012	2012	Japan	NA1	Saikusa, M. et al.
LC365888	HRSV/Yokohama.JPN/P6547/2012	2012	Japan	NA1	Saikusa, M. et al.
LC365889	HRSV/Yokohama.JPN/P6548/2012	2012	Japan	NA1	Saikusa, M. et al.
LC365893	HRSV/Yokohama.JPN/P6577/2012	2012	Japan	NA1	Saikusa, M. et al.
LC365894	HRSV/Yokohama.JPN/P6579/2012	2012	Japan	NA1	Saikusa, M. et al.
LC365895	HRSV/Yokohama.JPN/P6580/2012	2012	Japan	NA1	Saikusa, M. et al.
LC365896	HRSV/Yokohama.JPN/P6581/2012	2012	Japan	NA1	Saikusa, M. et al.
LC365897	HRSV/Yokohama.JPN/P6582/2012	2012	Japan	NA1	Saikusa, M. et al.
LC365898	HRSV/Yokohama.JPN/P6587/2012	2012	Japan	NA1	Saikusa, M. et al.
LC365899	HRSV/Yokohama.JPN/P6605/2012	2012	Japan	NA1	Saikusa, M. et al.
LC365900	HRSV/Yokohama.JPN/P6606/2012	2012	Japan	NA1	Saikusa, M. et al.
LC365901	HRSV/Yokohama.JPN/P6621/2012	2012	Japan	NA1	Saikusa, M. et al.
LC365902	HRSV/Yokohama.JPN/P6631/2012	2012	Japan	NA1	Saikusa, M. et al.
LC365903	HRSV/Yokohama.JPN/P6658/2012	2012	Japan	NA1	Saikusa, M. et al.
LC365904	HRSV/Yokohama.JPN/P6669/2012	2012	Japan	NA1	Saikusa, M. et al.
LC365905	HRSV/Yokohama.JPN/P6680/2012	2012	Japan	NA1	Saikusa, M. et al.
LC365906	HRSV/Yokohama.JPN/P6681/2012	2012	Japan	NA1	Saikusa, M. et al.
LC365907	HRSV/Yokohama.JPN/P6696/2012	2012	Japan	NA1	Saikusa, M. et al.
LC365908	HRSV/Yokohama.JPN/P6694/2013	2013	Japan	NA1	Saikusa, M. et al.
LC365909	HRSV/Yokohama.JPN/P6768/2013	2013	Japan	NA1	Saikusa, M. et al.
LC365910	HRSV/Yokohama.JPN/P6775/2013	2013	Japan	NA1	Saikusa, M. et al.
LC365911	HRSV/Yokohama.JPN/P6798/2013	2013	Japan	NA1	Saikusa, M. et al.
LC365912	HRSV/Yokohama.JPN/P6812/2013	2013	Japan	NA1	Saikusa, M. et al.
LC365914	HRSV/Yokohama.JPN/P6822/2013	2013	Japan	NA1	Saikusa, M. et al.

LC365915	HRSV/Yokohama.JPN/P6892/2013	2013	Japan	NA1	Saikusa, M. et al.
LC365916	HRSV/Yokohama.JPN/P6949/2013	2013	Japan	NA1	Saikusa, M. et al.
LC367132	HRSV/Yokohama.JPN/P7052/2013	2013	Japan	NA1	Saikusa, M. et al.
LC367133	HRSV/Yokohama.JPN/P7087/2013	2013	Japan	NA1	Saikusa, M. et al.
LC367135	HRSV/Yokohama.JPN/P7111/2013	2013	Japan	NA1	Saikusa, M. et al.
LC367136	HRSV/Yokohama.JPN/P7112/2013	2013	Japan	NA1	Saikusa, M. et al.
LC367137	HRSV/Yokohama.JPN/P7134/2013	2013	Japan	NA1	Saikusa, M. et al.
LC367138	HRSV/Yokohama.JPN/P7136/2013	2013	Japan	NA1	Saikusa, M. et al.
LC367139	HRSV/Yokohama.JPN/P7137/2013	2013	Japan	NA1	Saikusa, M. et al.
LC367140	HRSV/Yokohama.JPN/P7142/2013	2013	Japan	NA1	Saikusa, M. et al.

Supplementary Table S1 (continued). Strains used in this study.

GenBank accession No.	Strain	Collection year	Country	Genotype	References or authorship
LC367141	HRSV/Yokohama.JPN/P7154/2013	2013	Japan	NA1	Saikusa, M. et al.
LC367142	HRSV/Yokohama.JPN/P7158/2013	2013	Japan	NA1	Saikusa, M. et al.
LC367144	HRSV/Yokohama.JPN/P7190/2013	2013	Japan	NA1	Saikusa, M. et al.
LC367145	HRSV/Yokohama.JPN/P7194/2013	2013	Japan	NA1	Saikusa, M. et al.
LC367146	HRSV/Yokohama.JPN/P7204/2013	2013	Japan	NA1	Saikusa, M. et al.
LC367147	HRSV/Yokohama.JPN/P7216/2013	2013	Japan	NA1	Saikusa, M. et al.
LC367148	HRSV/Yokohama.JPN/P7218/2013	2013	Japan	NA1	Saikusa, M. et al.
LC367149	HRSV/Yokohama.JPN/P7226/2013	2013	Japan	NA1	Saikusa, M. et al.
LC367150	HRSV/Yokohama.JPN/P7232/2013	2013	Japan	NA1	Saikusa, M. et al.
LC367151	HRSV/Yokohama.JPN/P7233/2013	2013	Japan	NA1	Saikusa, M. et al.
LC367152	HRSV/Yokohama.JPN/P7239/2013	2013	Japan	NA1	Saikusa, M. et al.
LC367153	HRSV/Yokohama.JPN/P7469/2014	2014	Japan	NA1	Saikusa, M. et al.
LC367161	HRSV/Yokohama.JPN/P7620/2014	2014	Japan	NA1	Saikusa, M. et al.
LC367162	HRSV/Yokohama.JPN/P7623/2014	2014	Japan	NA1	Saikusa, M. et al.
LC367163	HRSV/Yokohama.JPN/P7624/2014	2014	Japan	NA1	Saikusa, M. et al.
LC367164	HRSV/Yokohama.JPN/P7629/2014	2014	Japan	NA1	Saikusa, M. et al.
LC367166	HRSV/Yokohama.JPN/P7683/2014	2014	Japan	NA1	Saikusa, M. et al.
LC367168	HRSV/Yokohama.JPN/P7694/2014	2014	Japan	NA1	Saikusa, M. et al.
LC367172	HRSV/Yokohama.JPN/P7714/2014	2014	Japan	NA1	Saikusa, M. et al.
LC367173	HRSV/Yokohama.JPN/P7718/2014	2014	Japan	NA1	Saikusa, M. et al.
LC367174	HRSV/Yokohama.JPN/P7744/2014	2014	Japan	NA1	Saikusa, M. et al.
LC367175	HRSV/Yokohama.JPN/P7745/2014	2014	Japan	NA1	Saikusa, M. et al.
LC367176	HRSV/Yokohama.JPN/P7764/2015	2015	Japan	NA1	Saikusa, M. et al.
LC367177	HRSV/Yokohama.JPN/P7785/2015	2015	Japan	NA1	Saikusa, M. et al.
LC367178	HRSV/Yokohama.JPN/P7795/2015	2015	Japan	NA1	Saikusa, M. et al.
LC367179	HRSV/Yokohama.JPN/P7904/2015	2015	Japan	NA1	Saikusa, M. et al.
LC367182	HRSV/Yokohama.JPN/P8106/2015	2015	Japan	NA1	Saikusa, M. et al.
LC367183	HRSV/Yokohama.JPN/P8109/2015	2015	Japan	NA1	Saikusa, M. et al.
LC367184	HRSV/Yokohama.JPN/P8112/2015	2015	Japan	NA1	Saikusa, M. et al.
LC367186	HRSV/Yokohama.JPN/P8139/2015	2015	Japan	NA1	Saikusa, M. et al.
LC367188	HRSV/Yokohama.JPN/P8153/2015	2015	Japan	NA1	Saikusa, M. et al.
LC367189	HRSV/Yokohama.JPN/P8158/2015	2015	Japan	NA1	Saikusa, M. et al.
LC367190	HRSV/Yokohama.JPN/P8176/2015	2015	Japan	NA1	Saikusa, M. et al.
LC367191	HRSV/Yokohama.JPN/P8188/2015	2015	Japan	NA1	Saikusa, M. et al.
LC367192	HRSV/Yokohama.JPN/P8197/2015	2015	Japan	NA1	Saikusa, M. et al.
LC367193	HRSV/Yokohama.JPN/P8204/2015	2015	Japan	NA1	Saikusa, M. et al.
LC367194	HRSV/Yokohama.JPN/P8217/2015	2015	Japan	NA1	Saikusa, M. et al.
LC367195	HRSV/Yokohama.JPN/P8219/2015	2015	Japan	NA1	Saikusa, M. et al.
LC367196	HRSV/Yokohama.JPN/P8237/2016	2016	Japan	NA1	Saikusa, M. et al.
LC367197	HRSV/Yokohama.JPN/P8238/2016	2016	Japan	NA1	Saikusa, M. et al.
LC367198	HRSV/Yokohama.JPN/P8242/2016	2016	Japan	NA1	Saikusa, M. et al.
LC367199	HRSV/Yokohama.JPN/I927/2016	2016	Japan	NA1	Saikusa, M. et al.
LC367200	HRSV/Yokohama.JPN/P8361/2016	2016	Japan	NA1	Saikusa, M. et al.
LC367202	HRSV/Yokohama.JPN/P8392/2016	2016	Japan	NA1	Saikusa, M. et al.
LC367203	HRSV/Yokohama.JPN/P8565/2016	2016	Japan	NA1	Saikusa, M. et al.
LC367206	HRSV/Yokohama.JPN/P8575/2016	2016	Japan	NA1	Saikusa, M. et al.
LC367207	HRSV/Yokohama.JPN/P8585/2016	2016	Japan	NA1	Saikusa, M. et al.
LC367208	HRSV/Yokohama.JPN/P8594/2016	2016	Japan	NA1	Saikusa, M. et al.
LC367210	HRSV/Yokohama.JPN/P8601/2016	2016	Japan	NA1	Saikusa, M. et al.

LC367211	HRSV/Yokohama.JPN/P8609/2016	2016	Japan	NA1	Saikusa, M. et al.
LC367212	HRSV/Yokohama.JPN/P8670/2016	2016	Japan	NA1	Saikusa, M. et al.
LC367213	HRSV/Yokohama.JPN/P8673/2016	2016	Japan	NA1	Saikusa, M. et al.
LC367214	HRSV/Yokohama.JPN/P8697/2016	2016	Japan	NA1	Saikusa, M. et al.
LC367217	HRSV/Yokohama.JPN/P8898/2017	2017	Japan	NA1	Saikusa, M. et al.
LC367218	HRSV/Yokohama.JPN/P8936/2017	2017	Japan	NA1	Saikusa, M. et al.
LC367219	HRSV/Yokohama.JPN/P8980/2017	2017	Japan	NA1	Saikusa, M. et al.
LC377910	TB5_CA-14-0345	2014	Philippines	NA1	22
LC377911	TB1_KW-14-0022	2014	Philippines	NA1	22
LC474556	RSV/A/NIID/2347/14	2014	USA	NA1	Shirato, K. et al.

Supplementary Table S1 (continued). Strains used in this study.

GenBank accession No.	Strain	Collection year	Country	Genotype	References or authorship
LC474557	RSV/A/NIID/2367/14	2014	USA	NA1	Shirato, K. et al.
LC474558	RSV/A/NIID/2370/14	2014	USA	NA1	Shirato, K. et al.
LC530050	sugckmmaht NGO3	2019	Japan	NA1	Takeuchi, S. et al.
MF001041	6A5	2015	USA	NA1	Greninger, A.L. et al.
MF001043	6A7	2015	USA	NA1	Greninger, A.L. et al.
MF001047	6O1	2015	USA	NA1	Greninger, A.L. et al.
MF001050	8P3	2015	USA	NA1	Greninger, A.L. et al.
MF001051	8P4	2015	USA	NA1	Greninger, A.L. et al.
MF001052	8P9	2015	USA	NA1	Greninger, A.L. et al.
MF001053	8Q1	2015	USA	NA1	Greninger, A.L. et al.
MF001054	8Q3	2015	USA	NA1	Greninger, A.L. et al.
MF001057	8S5	2015	USA	NA1	Greninger, A.L. et al.
MF361899	MAD/GM2_2/12	2012	Spain	NA1	2
MF361901	MAD/GM2_13/12	2012	Spain	NA1	2
MF361902	MAD/GM2_14/12	2012	Spain	NA1	2
MF445909	FJ121101-16	2012	China	NA1	Su, Y. et al.
MF445910	FJ111121-12	2011	China	NA1	Su, Y. et al.
MF445912	FJ130211-06	2013	China	NA1	Su, Y. et al.
MF445913	FJ131204-24	2013	China	NA1	Su, Y. et al.
MF445915	FJ141121-24	2014	China	NA1	Su, Y. et al.
MF445917	FJ150219-36	2015	China	NA1	Su, Y. et al.
MF445918	FJ150223-25	2015	China	NA1	Su, Y. et al.
MF445919	FJ111207-05	2011	China	NA1	Su, Y. et al.
MF445920	FJ121125-58	2012	China	NA1	Su, Y. et al.
MF445922	FJ130314-07	2013	China	NA1	Su, Y. et al.
MF445923	FJ130314-08	2013	China	NA1	Su, Y. et al.
MF445924	FJ131007-09	2013	China	NA1	Su, Y. et al.
MF445925	FJ131127-14	2013	China	NA1	Su, Y. et al.
MF445926	FJ120326-15	2012	China	NA1	Su, Y. et al.
MF445928	FJ141202-29	2014	China	NA1	Su, Y. et al.
MF445929	FJ150112-24	2015	China	NA1	Su, Y. et al.
MF445930	FJ150117-62	2015	China	NA1	Su, Y. et al.
MF445931	FJ150204-34	2015	China	NA1	Su, Y. et al.
MF445932	FJ150319-13	2015	China	NA1	Su, Y. et al.
MF445933	FJ131212-17	2013	China	NA1	Su, Y. et al.
MF445934	FJ120113-07	2012	China	NA1	Su, Y. et al.
MF445935	FJ131128-19	2013	China	NA1	Su, Y. et al.
MF445936	FJ131222-27	2013	China	NA1	Su, Y. et al.
MF445937	FJ140104-09	2014	China	NA1	Su, Y. et al.
MF445938	FJ140112-12	2014	China	NA1	Su, Y. et al.
MF445939	FJ141121-25	2014	China	NA1	Su, Y. et al.
MF445940	FJ141209-31	2014	China	NA1	Su, Y. et al.
MF445951	FJ120121-36	2012	China	NA1	Su, Y. et al.
MF445952	FJ111227-14	2011	China	NA1	Su, Y. et al.
MF445953	FJ120108-05	2012	China	NA1	Su, Y. et al.
MF445954	FJ120121-14	2012	China	NA1	Su, Y. et al.
MF445955	FJ120129-13	2012	China	NA1	Su, Y. et al.
MF445956	FJ120202-24	2012	China	NA1	Su, Y. et al.
MF445957	FJ101227-13	2010	China	NA1	Su, Y. et al.

MF445958	FJ110101-11	2011	China	NA1	Su, Y. et al.
MF445959	FJ110328-24A	2011	China	NA1	Su, Y. et al.
MF445960	FJ110101-46	2011	China	NA1	Su, Y. et al.
MF445961	FJ110111-23	2011	China	NA1	Su, Y. et al.
MF445962	FJ110305-45	2011	China	NA1	Su, Y. et al.
MF445963	FJ111026-32	2011	China	NA1	Su, Y. et al.
MF445964	FJ111114-24	2011	China	NA1	Su, Y. et al.
MF614946	BJ/40180	2012	China	NA1	Cui, G. et al.
MF614947	BJ/45117	2013	China	NA1	Cui, G. et al.
MF978510	RSVA-CAP-1/BJ/2014	2014	China	NA1	12

Supplementary Table S1 (continued). Strains used in this study.

GenBank accession No.	Strain	Collection year	Country	Genotype	References or authorship
MF978511	RSVA-CAP-8/BJ/2014	2015	China	NA1	12
MF978513	RSVA-CAP-10/BJ/2014	2014	China	NA1	12
MF978514	RSVA-CAP-11/BJ/2014	2014	China	NA1	12
MF978515	RSVA-CAP-14/BJ/2014	2014	China	NA1	12
MF978516	RSVA-CAP-22/BJ/2014	2014	China	NA1	12
MF978517	RSVA-CAP-30/BJ/2015	2015	China	NA1	12
MF978518	RSVA-CAP-31/BJ/2015	2015	China	NA1	12
MF978519	RSVA-CAP-33/BJ/2015	2015	China	NA1	12
MF978520	RSVA-CAP-42/BJ/2015	2015	China	NA1	12
MF978521	RSVA-CAP-43/BJ/2015	2015	China	NA1	12
MF978522	RSVA-CAP-54/BJ/2015	2015	China	NA1	12
MF978523	RSVA-CAP-56/BJ/2015	2015	China	NA1	12
MF978524	RSVA-CAP-61/BJ/2015	2015	China	NA1	12
MF978525	RSVA-CAP-66/BJ/2015	2015	China	NA1	12
MF978526	RSVA-CAP-67/BJ/2015	2015	China	NA1	12
MF978527	RSVA-CAP-92/BJ/2015	2015	China	NA1	12
MF978528	RSVA-CAP-132/BJ/2015	2015	China	NA1	12
MF978529	RSVA-CAP-234/BJ/2015	2015	China	NA1	12
MF978530	RSVA-CAP-250/BJ/2015	2015	China	NA1	12
MF978531	RSVA-CAP-255/BJ/2015	2015	China	NA1	12
MF978532	RSVA-CAP-328/BJ/2015	2015	China	NA1	12
MF978533	RSVA-CAP-356/BJ/2015	2015	China	NA1	12
MF978534	RSVA-CAP-398/BJ/2015	2015	China	NA1	12
MF978535	RSVA-CAP-402/BJ/2015	2015	China	NA1	12
MF978536	RSVA-CAP-405/BJ/2016	2016	China	NA1	12
MF978537	RSVB-CQ-115/CQ/2015	2015	China	NA1	12
MF978538	RSVB-CQ-235/CQ/2015	2015	China	NA1	12
MF978539	RSVA-GYYF-1/GZ/2015	2015	China	NA1	12
MF978540	RSVA-GYYF-3/GZ/2015	2015	China	NA1	12
MF978541	RSVA-GYYF-36/GZ/2015	2015	China	NA1	12
MF978542	RSVA-GYYF-55/GZ/2015	2015	China	NA1	12
MF978543	RSVA-GYYF-57/GZ/2015	2015	China	NA1	12
MF978544	RSVA-GYYF-91/GZ/2015	2015	China	NA1	12
MF978545	RSVA-GYYF-106/GZ/2016	2016	China	NA1	12
MF978546	RSVA-GYYF-148/GZ/2016	2016	China	NA1	12
MF978547	RSVA-GZFE-9/GZ/2015	2015	China	NA1	12
MF978548	RSVA-GZFE-22/GZ/2015	2015	China	NA1	12
MF978549	RSVA-GZFE-32/GZ/2015	2015	China	NA1	12
MF978550	RSVA-GZFE-34/GZ/2015	2015	China	NA1	12
MF978551	RSVA-GZFE-114/GZ/2015	2015	China	NA1	12
MF978552	RSVA-GZFE-128/GZ/2015	2015	China	NA1	12
MF978553	RSVA-GZFE-150/GZ/2015	2015	China	NA1	12
MF978554	RSVA-HB-80/HeB/2014	2014	China	NA1	12
MF978555	RSVA-HB-84/HeB/2014	2014	China	NA1	12
MF978556	RSVA-HB-88/HeB/2014	2014	China	NA1	12
MF978557	RSVA-HB-89/HeB/2014	2014	China	NA1	12
MF978558	RSVA-HB-97/HeB/2014	2014	China	NA1	12
MF978559	RSVA-HB-99/HeB/2014	2014	China	NA1	12
MF978561	RSVA-HB-102/HeB/2014	2014	China	NA1	12

MF978562	RSVA-HB-197/HeB/2015	2015	China	NA1	12
MF978563	RSVA-HB-216/HeB/2015	2015	China	NA1	12
MF978565	RSVA-HB-251/HeB/2015	2015	China	NA1	12
MF978566	RSVA-HB-263/HeB/2015	2015	China	NA1	12
MF978569	RSVA-HB-274/HeB/2015	2015	China	NA1	12
MF978570	RSVA-HB-276/HeB/2015	2015	China	NA1	12
MF978571	RSVA-HB-277/HeB/2015	2015	China	NA1	12
MF978572	RSVA-HB-279/HeB/2015	2015	China	NA1	12
MF978573	RSVA-HB-294/HeB/2016	2016	China	NA1	12
MF978574	RSVA-HB-297/HeB/2016	2016	China	NA1	12

Supplementary Table S1 (continued). Strains used in this study.

GenBank accession No.	Strain	Collection year	Country	Genotype	References or authorship
MF978575	RSVA-HB-307/HeB/2016	2016	China	NA1	12
MF978576	RSVA-HB-312/HeB/2016	2016	China	NA1	12
MF978578	RSVA-WZ-6/ZJ/2015	2015	China	NA1	12
MF978579	RSVA-WZ-10/ZJ/2015	2015	China	NA1	12
MF978581	RSVA-WZ-16/ZJ/2015	2015	China	NA1	12
MF978582	RSVA-WZ-17/ZJ/2015	2015	China	NA1	12
MF978583	RSVA-WZ-19/ZJ/2015	2015	China	NA1	12
MF978584	RSVA-WZ-20/ZJ/2015	2015	China	NA1	12
MF978585	RSVA-WZ-23/ZJ/2015	2015	China	NA1	12
MF978586	RSVA-WZ-26/ZJ/2015	2015	China	NA1	12
MF978587	RSVA-WZ-28/ZJ/2015	2015	China	NA1	12
MF978589	RSVA-WZ-33/ZJ/2015	2015	China	NA1	12
MF978591	RSVA-WZ-38/ZJ/2015	2015	China	NA1	12
MF978592	RSVA-WZ-40/ZJ/2015	2015	China	NA1	12
MF978595	RSVA-WZ-44/ZJ/2015	2015	China	NA1	12
MF978596	RSVA-WZ-47/ZJ/2015	2015	China	NA1	12
MF978598	RSVA-WZ-52/ZJ/2015	2015	China	NA1	12
MF978599	RSVA-WZ-53/ZJ/2015	2015	China	NA1	12
MF978600	RSVA-WZ-57/ZJ/2015	2015	China	NA1	12
MF978601	RSVA-WZ-60/ZJ/2015	2015	China	NA1	12
MF978603	RSVA-WZ-64/ZJ/2015	2015	China	NA1	12
MF978605	RSVA-WZ-68/ZJ/2015	2015	China	NA1	12
MF978606	RSVA-WZ-72/ZJ/2015	2015	China	NA1	12
MF978607	RSVA-WZ-73/ZJ/2015	2015	China	NA1	12
MF978608	RSVA-WZ-76/ZJ/2015	2015	China	NA1	12
MF978609	RSVA-WZ-79/ZJ/2015	2015	China	NA1	12
MF978610	RSVA-WZ-82/ZJ/2015	2015	China	NA1	12
MF978611	RSVA-WZ-84/ZJ/2015	2015	China	NA1	12
MF978612	RSVA-WZ-85/ZJ/2015	2015	China	NA1	12
MF978613	RSVA-WZ-92/ZJ/2015	2015	China	NA1	12
MF978614	RSVA-WZ-94/ZJ/2015	2015	China	NA1	12
MF978615	RSVA-WZ-101/ZJ/2015	2015	China	NA1	12
MF978617	RSVA-WZ-109/ZJ/2015	2015	China	NA1	12
MF978618	RSVA-WZ-113/ZJ/2015	2015	China	NA1	12
MF978619	RSVA-WZ-114/ZJ/2015	2015	China	NA1	12
MF978620	RSVA-WZ-117/ZJ/2015	2015	China	NA1	12
MF978621	RSVA-WZ-122/ZJ/2015	2015	China	NA1	12
MF978622	RSVA-WZ-123/ZJ/2015	2015	China	NA1	12
MF978623	RSVA-WZ-127/ZJ/2015	2015	China	NA1	12
MF978624	RSVA-WZ-128/ZJ/2015	2015	China	NA1	12
MF978626	RSVA-WZ-133/ZJ/2015	2015	China	NA1	12
MF978627	RSVA-WZ-135/ZJ/2015	2015	China	NA1	12
MF978628	RSVA-WZ-154/ZJ/2015	2015	China	NA1	12
MF978629	RSVA-WZ-160/ZJ/2015	2015	China	NA1	12
MF978630	RSVA-WZ-168/ZJ/2015	2015	China	NA1	12
MF978631	RSVA-WZ-169/ZJ/2015	2015	China	NA1	12
MF978633	RSVA-WZ-182/ZJ/2015	2015	China	NA1	12
MF978634	RSVB-WZ-183/ZJ/2015	2015	China	NA1	12
MF978638	RSVA-WZ-192/ZJ/2015	2015	China	NA1	12

MF978639	RSVA-WZ-193/ZJ/2015	2015	China	NA1	12
MF978640	RSVA-WZ-195/ZJ/2015	2015	China	NA1	12
MF978642	RSVA-WZ-207/ZJ/2015	2015	China	NA1	12
MF978643	RSVA-WZ-208/ZJ/2015	2015	China	NA1	12
MF978644	RSVA-WZ-213/ZJ/2015	2015	China	NA1	12
MF978645	RSVA-WZ-215/ZJ/2015	2015	China	NA1	12
MF978646	RSVA-WZ-217/ZJ/2015	2015	China	NA1	12
MF978647	RSVA-WZ-220/ZJ/2015	2015	China	NA1	12
MF978648	RSVA-WZ-221/ZJ/2015	2015	China	NA1	12
MF978649	RSVA-WZ-232/ZJ/2015	2015	China	NA1	12

Supplementary Table S1 (continued). Strains used in this study.

GenBank accession No.	Strain	Collection year	Country	Genotype	References or authorship
MF978651	RSVA-WZ-285/ZJ/2015	2015	China	NA1	12
MF978652	RSVA-WZ-287/ZJ/2015	2015	China	NA1	12
MF978653	RSVA-WZ-288/ZJ/2015	2015	China	NA1	12
MF978654	RSVA-WZ-336/ZJ/2015	2015	China	NA1	12
MF978655	RSVA-WZ-348/ZJ/2015	2015	China	NA1	12
MF978656	RSVA-WZ-351/ZJ/2015	2015	China	NA1	12
MF978657	RSVA-WZ-358/ZJ/2015	2015	China	NA1	12
MF978658	RSVA-WZ-407/ZJ/2015	2015	China	NA1	12
MF978659	RSVA-WZ-441/ZJ/2015	2015	China	NA1	12
MF978660	RSVA-WZ-460/ZJ/2015	2015	China	NA1	12
MF978661	RSVA-WZ-466/ZJ/2015	2015	China	NA1	12
MF978663	RSVA-WZ-490/ZJ/2015	2015	China	NA1	12
MF978664	RSVA-WZ-492/ZJ/2015	2015	China	NA1	12
MF978665	RSVA-WZ-516/ZJ/2015	2015	China	NA1	12
MF978666	RSVA-WZ-536/ZJ/2015	2015	China	NA1	12
MF978667	RSVA-YC-161/NX/2015	2015	China	NA1	12
MF978668	RSVA-YC-177/NX/2015	2015	China	NA1	12
MF978669	RSVA-YC-187/NX/2015	2015	China	NA1	12
MF978670	RSVA-YC-188/NX/2015	2015	China	NA1	12
MF978671	RSVA-YC-195/NX/2015	2015	China	NA1	12
MF978672	RSVA-YC-200/NX/2015	2015	China	NA1	12
MF978673	RSVA-YC-202/NX/2015	2015	China	NA1	12
MF978674	RSVA-ZJ-276/ZJ/2015	2015	China	NA1	12
MF978675	RSVA-ZJ-288/ZJ/2015	2015	China	NA1	12
MF978676	RSVA-ZJ-290/ZJ/2015	2015	China	NA1	12
MF978677	RSVA-ZJ-296/ZJ/2015	2015	China	NA1	12
MF978678	RSVA-ZJ-298/ZJ/2015	2015	China	NA1	12
MF978679	RSVA-ZJ-299/ZJ/2015	2015	China	NA1	12
MF978680	RSVA-ZJ-301/ZJ/2015	2015	China	NA1	12
MF978681	RSVA-ZJ-302/ZJ/2015	2015	China	NA1	12
MF978682	RSVA-ZJ-303/ZJ/2015	2015	China	NA1	12
MF978683	RSVA-ZJ-310/ZJ/2015	2015	China	NA1	12
MF978684	RSVA-ZJ-315/ZJ/2015	2015	China	NA1	12
MF978685	RSVA-ZJ-323/ZJ/2015	2015	China	NA1	12
MF978686	RSVA-ZJ-325/ZJ/2015	2015	China	NA1	12
MF978687	RSVA-ZJ-332/ZJ/2015	2015	China	NA1	12
MF978689	RSVA-ZJ-348/ZJ/2015	2015	China	NA1	12
MF978691	RSVA-ZJ-369/ZJ/2015	2015	China	NA1	12
MF978692	RSVA-ZJ-398/ZJ/2015	2015	China	NA1	12
MG431254	STA836	2010	Brazil	NA1	Di Paola, N. et al.
MG431255	STA839	2010	Brazil	NA1	Di Paola, N. et al.
MG773271	HRSV/A/BuenosAires/ARG/011/2016	2016	Argentina	NA1	23
MG793382	RSVA/Lebanon/14LJF289/2015	2015	Lebanon	NA1	Ezzeddine, A.M.
MG813979	A-TX-009b-2012-WGS	2012	USA	NA1	Piedra, F.-A. et al.
MG813983	A-TX-032b-2012-WGS	2012	USA	NA1	Piedra, F.-A. et al.
MG813985	A-TX-010b-2014-WGS	2014	USA	NA1	Piedra, F.-A. et al.
MG813987	A-TX-012b-2015-WGS	2015	USA	NA1	Piedra, F.-A. et al.
MG813991	A-TX-018b-2012-WGS	2012	USA	NA1	Piedra, F.-A. et al.
MG839543	HRSV/A/BuenosAires/ARG/012/2015	2015	Argentina	NA1	23

MH181878	KEN/KILIFI/WGS/1022_28/12/2011	2011	Kenya	NA1	24
MH181881	KEN/KILIFI/WGS/1028_02/03/2012	2012	Kenya	NA1	24
MH181885	KEN/KILIFI/WGS/1039_25/03/2012	2012	Kenya	NA1	24
MH181888	KEN/KILIFI/WGS/1047_15/04/2012	2012	Kenya	NA1	24
MH181889	KEN/KILIFI/WGS/1048_16/04/2012	2012	Kenya	NA1	24
MH181890	KEN/KILIFI/WGS/1049_17/04/2012	2012	Kenya	NA1	24
MH181891	KEN/KILIFI/WGS/1050_17/04/2012	2012	Kenya	NA1	24
MH181892	KEN/KILIFI/WGS/1052_25/04/2012	2012	Kenya	NA1	24
MH181893	KEN/KILIFI/WGS/1064_24/06/2012	2012	Kenya	NA1	24
MH181894	KEN/KILIFI/WGS/1067_30/06/2012	2012	Kenya	NA1	24

Supplementary Table S1 (continued). Strains used in this study.

GenBank accession No.	Strain	Collection year	Country	Genotype	References or authorship
MH181895	KEN/KILIFI/WGS/1101_25/11/2012	2012	Kenya	NA1	24
MH181897	KEN/KILIFI/WGS/1138_14/01/2013	2013	Kenya	NA1	24
MH181898	KEN/KILIFI/WGS/1146_03/02/2013	2013	Kenya	NA1	24
MH181901	KEN/KILIFI/WGS/1155_13/03/2013	2013	Kenya	NA1	24
MH181902	KEN/KILIFI/WGS/1159_24/05/2013	2013	Kenya	NA1	24
MH181904	KEN/KILIFI/WGS/1169_07/11/2013	2013	Kenya	NA1	24
MH181905	KEN/KILIFI/WGS/1215_15/04/2014	2014	Kenya	NA1	24
MH181906	KEN/KILIFI/WGS/1314_31/03/2015	2015	Kenya	NA1	24
MH181907	KEN/KILIFI/WGS/1322_17/04/2014	2015	Kenya	NA1	24
MH181910	KEN/KILIFI/WGS/1033_09/03/2012	2012	Kenya	NA1	24
MH181911	KEN/KILIFI/WGS/1034_11/03/2012	2012	Kenya	NA1	24
MH181915	KEN/KILIFI/WGS/1040_26/03/2012	2012	Kenya	NA1	24
MH181916	KEN/KILIFI/WGS/1041_29/03/2012	2012	Kenya	NA1	24
MH181918	KEN/KILIFI/WGS/1044_10/04/2012	2012	Kenya	NA1	24
MH181920	KEN/KILIFI/WGS/1053_26/04/2012	2012	Kenya	NA1	24
MH181925	KEN/KILIFI/WGS/1060_11/06/2012	2012	Kenya	NA1	24
MH181928	KEN/KILIFI/WGS/1063_18/06/2012	2012	Kenya	NA1	24
MH181929	KEN/KILIFI/WGS/1066_28/06/2012	2012	Kenya	NA1	24
MH181931	KEN/KILIFI/WGS/1070_23/07/2012	2012	Kenya	NA1	24
MH181933	KEN/KILIFI/WGS/1075_27/10/2012	2012	Kenya	NA1	24
MH181938	KEN/KILIFI/WGS/1080_05/11/2012	2012	Kenya	NA1	24
MH181940	KEN/KILIFI/WGS/1082_10/11/2012	2012	Kenya	NA1	24
MH181941	KEN/KILIFI/WGS/1083_12/11/2012	2012	Kenya	NA1	24
MH181943	KEN/KILIFI/WGS/1086_15/11/2012	2012	Kenya	NA1	24
MH181950	KEN/KILIFI/WGS/1093_23/11/2012	2012	Kenya	NA1	24
MH181953	KEN/KILIFI/WGS/1096_24/11/2012	2012	Kenya	NA1	24
MH181954	KEN/KILIFI/WGS/1097_24/11/2012	2012	Kenya	NA1	24
MH181959	KEN/KILIFI/WGS/1103_27/11/2012	2012	Kenya	NA1	24
MH181960	KEN/KILIFI/WGS/1104_27/11/2012	2012	Kenya	NA1	24
MH181967	KEN/KILIFI/WGS/1113_03/12/2012	2012	Kenya	NA1	24
MH181969	KEN/KILIFI/WGS/1115_04/12/2012	2012	Kenya	NA1	24
MH181971	KEN/KILIFI/WGS/1118_12/12/2012	2012	Kenya	NA1	24
MH181972	KEN/KILIFI/WGS/1119_15/12/2012	2012	Kenya	NA1	24
MH181973	KEN/KILIFI/WGS/1120_19/12/2012	2012	Kenya	NA1	24
MH181974	KEN/KILIFI/WGS/1121_21/12/2012	2012	Kenya	NA1	24
MH181976	KEN/KILIFI/WGS/1124_24/12/2012	2012	Kenya	NA1	24
MH181977	KEN/KILIFI/WGS/1125_26/12/2012	2012	Kenya	NA1	24
MH181980	KEN/KILIFI/WGS/1129_08/01/2013	2013	Kenya	NA1	24
MH181982	KEN/KILIFI/WGS/1132_11/01/2013	2013	Kenya	NA1	24
MH181984	KEN/KILIFI/WGS/1134_12/01/2013	2013	Kenya	NA1	24
MH181985	KEN/KILIFI/WGS/1140_17/01/2013	2013	Kenya	NA1	24
MH181987	KEN/KILIFI/WGS/1142_21/01/2013	2013	Kenya	NA1	24
MH181988	KEN/KILIFI/WGS/1144_24/01/2013	2013	Kenya	NA1	24
MH181989	KEN/KILIFI/WGS/1151_10/02/2013	2013	Kenya	NA1	24
MH181990	KEN/KILIFI/WGS/1153_12/03/2013	2013	Kenya	NA1	24
MH181991	KEN/KILIFI/WGS/1162_12/10/2013	2013	Kenya	NA1	24
MH181993	KEN/KILIFI/WGS/1166_28/10/2013	2013	Kenya	NA1	24
MH181996	KEN/KILIFI/WGS/1171_12/11/2013	2013	Kenya	NA1	24
MH181997	KEN/KILIFI/WGS/1173_19/11/2013	2013	Kenya	NA1	24

MH181998	KEN/Kilifi/WGS/1582_01/04/2014	2014	Kenya	NA1	24
MH181999	KEN/Kilifi/WGS/1583_13/04/2014	2014	Kenya	NA1	24
MH182001	KEN/KILIFI/WGS/1246_26/11/2014	2014	Kenya	NA1	24
MH182005	KEN/KILIFI/WGS/1251_06/12/2014	2014	Kenya	NA1	24
MH182010	KEN/KILIFI/WGS/1257_10/12/2014	2014	Kenya	NA1	24
MH182012	KEN/KILIFI/WGS/1260_14/12/2014	2014	Kenya	NA1	24
MH182016	KEN/KILIFI/WGS/1266_17/12/2014	2014	Kenya	NA1	24
MH182018	KEN/KILIFI/WGS/1269_17/12/2014	2014	Kenya	NA1	24
MH182019	KEN/KILIFI/WGS/1271_18/12/2014	2014	Kenya	NA1	24
MH182021	KEN/KILIFI/WGS/1274_21/12/2014	2014	Kenya	NA1	24

Supplementary Table S1 (continued). Strains used in this study.

GenBank accession No.	Strain	Collection year	Country	Genotype	References or authorship
MH182022	KEN/KILIFI/WGS/1275_22/12/2014	2014	Kenya	NA1	24
MH182023	KEN/KILIFI/WGS/1278_25/12/2014	2014	Kenya	NA1	24
MH182024	KEN/KILIFI/WGS/1279_27/12/2014	2014	Kenya	NA1	24
MH182025	KEN/KILIFI/WGS/1281_30/12/2014	2014	Kenya	NA1	24
MH182026	KEN/KILIFI/WGS/1282_31/12/2014	2014	Kenya	NA1	24
MH182027	KEN/KILIFI/WGS/1283_31/12/2014	2014	Kenya	NA1	24
MH182029	KEN/KILIFI/WGS/1286_04/01/2015	2015	Kenya	NA1	24
MH182030	KEN/KILIFI/WGS/1287_06/01/2015	2015	Kenya	NA1	24
MH182031	KEN/KILIFI/WGS/1288_07/01/2015	2015	Kenya	NA1	24
MH182032	KEN/KILIFI/WGS/1289_08/01/2015	2015	Kenya	NA1	24
MH182033	KEN/KILIFI/WGS/1290_08/01/2015	2015	Kenya	NA1	24
MH182035	KEN/KILIFI/WGS/1293_24/01/2015	2015	Kenya	NA1	24
MH182036	KEN/KILIFI/WGS/1295_27/01/2015	2015	Kenya	NA1	24
MH182037	KEN/KILIFI/WGS/1296_29/01/2015	2015	Kenya	NA1	24
MH182038	KEN/KILIFI/WGS/1298_31/01/2015	2015	Kenya	NA1	24
MH182039	KEN/KILIFI/WGS/1299_04/02/2015	2015	Kenya	NA1	24
MH182040	KEN/KILIFI/WGS/1301_08/02/2015	2015	Kenya	NA1	24
MH182041	KEN/KILIFI/WGS/1302_13/02/2015	2015	Kenya	NA1	24
MH182042	KEN/KILIFI/WGS/1305_21/02/2015	2015	Kenya	NA1	24
MH182046	KEN/KILIFI/WGS/1311_21/03/2015	2015	Kenya	NA1	24
MH182047	KEN/KILIFI/WGS/1312_22/03/2015	2015	Kenya	NA1	24
MH182048	KEN/KILIFI/WGS/1313_27/03/2015	2015	Kenya	NA1	24
MH182050	KEN/KILIFI/WGS/1318_08/04/2015	2015	Kenya	NA1	24
MH182051	KEN/KILIFI/WGS/1320_12/04/2015	2015	Kenya	NA1	24
MH182052	KEN/KILIFI/WGS/1321_17/04/2015	2015	Kenya	NA1	24
MH182054	KEN/Kilifi/WGS/1585_17/02/2016	2016	Kenya	NA1	24
MH182055	KEN/Kilifi/WGS/1586_28/01/2016	2016	Kenya	NA1	24
MH182056	KEN/Kilifi/WGS/1587_08/02/2016	2016	Kenya	NA1	24
MH182057	KEN/Kilifi/WGS/1588_29/03/2016	2016	Kenya	NA1	24
MH182058	KEN/Kilifi/WGS/1589_26/01/2016	2016	Kenya	NA1	24
MH182059	KEN/Kilifi/WGS/1590_01/02/2016	2016	Kenya	NA1	24
MH182060	KEN/Kilifi/WGS/1591_18/01/2016	2016	Kenya	NA1	24
MH182061	KEN/Kilifi/WGS/1592_07/04/2016	2016	Kenya	NA1	24
MH187282	RSVs/Tehran.IRN/A/53.15/2 [ON1]	2015	Iran	NA1	Salimi, V. et al.
MH187283	RSVs/Tehran.IRN/A/53.15/4 [ON1]	2015	Iran	NA1	Salimi, V. et al.
MH187284	RSVs/Tehran.IRN/A/1.16/1 [ON1]	2016	Iran	NA1	Salimi, V. et al.
MH187285	RSVs/Tehran.IRN/A/1.16/8 [ON1]	2016	Iran	NA1	Salimi, V. et al.
MH187286	RSVs/Tehran.IRN/A/1.16/11 [ON1]	2016	Iran	NA1	Salimi, V. et al.
MH187287	RSV/Tehran.IRN/A/1.16/12 [ON1]	2016	Iran	NA1	Salimi, V. et al.
MH187288	RSVs/Tehran.IRN/A/1.16/13 [ON1]	2016	Iran	NA1	Salimi, V. et al.
MH187289	RSVs/Tehran.IRN/A/2.16/1 [ON1]	2016	Iran	NA1	Salimi, V. et al.
MH187290	RSVs/Tehran.IRN/A/2.16/2 [ON1]	2016	Iran	NA1	Salimi, V. et al.
MH187291	RSVs/Tehran.IRN/A/2.16/3 [ON1]	2016	Iran	NA1	Salimi, V. et al.
MH187292	RSVs/Tehran.IRN/A/3.16/5 [ON1]	2016	Iran	NA1	Salimi, V. et al.
MH187293	RSVs/Tehran.IRN/A/3.16/8 [ON1]	2016	Iran	NA1	Salimi, V. et al.
MH187294	RSVs/Tehran.IRN/A/4.16/1 [ON1]	2016	Iran	NA1	Salimi, V. et al.
MH187295	RSVs/Tehran.IRN/A/4.16/2 [ON1]	2016	Iran	NA1	Salimi, V. et al.
MH187296	RSVs/Tehran.IRN/A/4.16/4 [ON1]	2016	Iran	NA1	Salimi, V. et al.
MH187297	RSVs/Tehran.IRN/A/4.16/5 [ON1]	2016	Iran	NA1	Salimi, V. et al.

MH187298	RSVs/Tehran.IRN/A/4.16/7 [ON1]	2016	Iran	NA1	Salimi, V. et al.
MH187299	RSVs/Tehran.IRN/A/4.16/10 [ON1]	2016	Iran	NA1	Salimi, V. et al.
MH187300	RSVs/Tehran.IRN/A/5.16/1 [ON1]	2016	Iran	NA1	Salimi, V. et al.
MH187301	RSVs/Tehran.IRN/A/6.16/1 [ON1]	2016	Iran	NA1	Salimi, V. et al.
MH187302	RSVs/Tehran.IRN/A/6.16/2 [ON1]	2016	Iran	NA1	Salimi, V. et al.
MH187303	RSVs/Tehran.IRN/A/6.16/3 [ON1]	2016	Iran	NA1	Salimi, V. et al.
MH187304	RSVs/Tehran.IRN/A/7.16/3 [ON1]	2016	Iran	NA1	Salimi, V. et al.
MH187305	RSVs/Tehran.IRN/A/7.16/2 [ON1]	2016	Iran	NA1	Salimi, V. et al.
MH187306	RSVs/Tehran.IRN/A/8.16/1 [ON1]	2016	Iran	NA1	Salimi, V. et al.
MH187307	RSVs/Tehran.IRN/A/8.16/2 [ON1]	2016	Iran	NA1	Salimi, V. et al.

Supplementary Table S1 (continued). Strains used in this study.

GenBank accession No.	Strain	Collection year	Country	Genotype	References or authorship
MH187308	RSVs/Tehran.IRN/A/11.16/1 [ON1]	2016	Iran	NA1	Salimi, V. et al.
MH279547	LZ170020448/17	2017	China	NA1	Qiao, R. et al.
MH290724	LZ170020467/17	2017	China	NA1	Qiao, R. et al.
MH383066	JF204	2014	Lebanon	NA1	Hamdan, F. et al.
MH447951	TH/CU91/2011	2011	Thailand	NA1	Thongpan, I.
MH447952	TH/C3208/2012	2012	Thailand	NA1	Thongpan, I.
MH447954	TH/CB135/2013	2013	Thailand	NA1	Thongpan, I.
MH447955	TH/B10625/2014	2014	Thailand	NA1	Thongpan, I.
MH447956	TH/B10806/2014	2014	Thailand	NA1	Thongpan, I.
MH447957	TH/B18177/2016	2016	Thailand	NA1	Thongpan, I.
MH447958	TH/B18247/2016	2016	Thailand	NA1	Thongpan, I.
MH447959	TH/B23576/2017	2017	Thailand	NA1	Thongpan, I.
MH447960	TH/B23964/2017	2017	Thailand	NA1	Thongpan, I.
MH760588	A/NSW/WM0301A/16	2016	Australia	NA1	13
MH760589	A/NSW/WM3629A/16	2016	Australia	NA1	13
MH760591	A/NSW/WM1734B/16	2016	Australia	NA1	13
MH760592	A/NSW/WM0276A/10	2010	Australia	NA1	13
MH760593	A/NSW/WM0231A/10	2010	Australia	NA1	13
MH760594	A/NSW/WM3702A/10	2010	Australia	NA1	13
MH760595	A/NSW/WM0484A/10	2010	Australia	NA1	13
MH760596	A/NSW/WM1135A/10	2010	Australia	NA1	13
MH760597	A/NSW/WM2192A/10	2010	Australia	NA1	13
MH760598	A/NSW/WM1654C/10	2010	Australia	NA1	13
MH760600	A/NSW/WM4173A/11	2011	Australia	NA1	13
MH760601	A/NSW/WM0597B/12	2012	Australia	NA1	13
MH760602	A/NSW/WM1236A/12	2012	Australia	NA1	13
MH760603	A/NSW/WM2829A/12	2012	Australia	NA1	13
MH760604	A/NSW/WM4460A/12	2012	Australia	NA1	13
MH760605	A/NSW/WM1837A/12	2012	Australia	NA1	13
MH760606	A/NSW/WM1008A/13	2013	Australia	NA1	13
MH760607	A/NSW/WM3453A/13	2013	Australia	NA1	13
MH760608	A/NSW/WM1112A/16	2016	Australia	NA1	13
MH760609	A/NSW/WM1514A/16	2016	Australia	NA1	13
MH760610	A/NSW/WM1585A/16	2016	Australia	NA1	13
MH760611	A/NSW/WM0467B/16	2016	Australia	NA1	13
MH760612	A/NSW/WM0166A/16	2016	Australia	NA1	13
MH760613	A/NSW/WM0672A/16	2016	Australia	NA1	13
MH760616	A/NSW/WM0371A/16	2016	Australia	NA1	13
MH760617	A/NSW/WM1681A/16	2016	Australia	NA1	13
MH760619	A/NSW/WM3743A/16	2016	Australia	NA1	13
MH760620	A/NSW/WM1053A/16	2016	Australia	NA1	13
MH760621	A/NSW/WM4241A/16	2016	Australia	NA1	13
MH760622	A/NSW/WM3992A/16	2016	Australia	NA1	13
MH760623	A/NSW/WM4754A/16	2016	Australia	NA1	13
MH760624	A/NSW/WM0737A/16	2016	Australia	NA1	13
MH760625	A/NSW/WM1079A/16	2016	Australia	NA1	13
MH760627	A/NSW/WM1045A/16	2016	Australia	NA1	13
MH760628	A/NSW/WM2369B/16	2016	Australia	NA1	13
MH760629	A/NSW/WM1991A/16	2016	Australia	NA1	13

MH760630	A/NSW/WM0778A/16	2016	Australia	NA1	13
MH760631	A/NSW/WM0879A/16	2016	Australia	NA1	13
MH760632	A/NSW/WM1554A/16	2016	Australia	NA1	13
MH760633	A/NSW/WM1825A/16	2016	Australia	NA1	13
MH760634	A/NSW/WM1254A/16	2016	Australia	NA1	13
MH760635	A/NSW/WM1670A/16	2016	Australia	NA1	13
MH760636	A/NSW/WM2797A/16	2016	Australia	NA1	13
MH760638	A/NSW/WM0230A/16	2016	Australia	NA1	13
MH760639	A/NSW/WM4313A/16	2016	Australia	NA1	13
MH760641	A/NSW/WM1473B/14	2014	Australia	NA1	13

Supplementary Table S1 (continued). Strains used in this study.

GenBank accession No.	Strain	Collection year	Country	Genotype	References or authorship
MH760642	A/NSW/WM2111B/14	2014	Australia	NA1	13
MH760643	A/NSW/WM3837A/14	2014	Australia	NA1	13
MH760644	A/NSW/WM2906A/14	2014	Australia	NA1	13
MH760645	A/NSW/WM0279A/14	2014	Australia	NA1	13
MH760646	A/NSW/WM0644C/14	2014	Australia	NA1	13
MH760647	A/NSW/WM3790A/15	2015	Australia	NA1	13
MH760648	A/NSW/WM2866A/15	2015	Australia	NA1	13
MH760649	A/NSW/WM1676B/15	2015	Australia	NA1	13
MH760651	A/NSW/WM0359B/15	2015	Australia	NA1	13
MK109773	RSVA/Homo sapiens/JOR/D3140/2011	2011	Jordan	NA1	Tan, G. et al.
MK109774	RSVA/Homo sapiens/JOR/C2101/2011	2011	Jordan	NA1	Tan, G. et al.
MK109775	RSVA/Homo sapiens/JOR/B1318/2011	2011	Jordan	NA1	Tan, G. et al.
MK109776	RSVA/Homo sapiens/JOR/D3159/2011	2011	Jordan	NA1	Tan, G. et al.
MK109777	RSVA/Homo sapiens/JOR/D3120/2011	2011	Jordan	NA1	Tan, G. et al.
MK109785	RSVA/Homo sapiens/JOR/D3055/2010	2010	Jordan	NA1	Tan, G. et al.
MK109787	RSVA/Homo sapiens/JOR/C2152/2011	2011	Jordan	NA1	Tan, G. et al.
MK167035	8L8	2008	USA	NA1	Greninger, A.L. et al.
MK167036	6B2	2006	USA	NA1	Greninger, A.L. et al.
MK749867	RSVA/Homo sapiens/NIC/IIIn_16_0242_01NT/2016	2016	Nicaragua	NA1	Tan, G. et al.
MK749884	RSVA/Homo sapiens/NIC/IIIn_16_0313_01NT/2016	2016	Nicaragua	NA1	Tan, G. et al.
MK749890	RSVA/Homo sapiens/NIC/IIIn_16_0270_01NT/2016	2016	Nicaragua	NA1	Tan, G. et al.
MK749893	RSVA/Homo sapiens/NIC/IIIn_16_0244_01NT/2016	2016	Nicaragua	NA1	Tan, G. et al.
MK749909	RSVA/Homo sapiens/NIC/IIIn_16_0267_01NT/2016	2016	Nicaragua	NA1	Tan, G. et al.
MK749911	RSVA/Homo sapiens/NIC/IIIn_16_0238_01NT/2016	2016	Nicaragua	NA1	Tan, G. et al.
MK749912	RSVA/Homo sapiens/NIC/IIIn_15_0256_01NT/2015	2015	Nicaragua	NA1	Tan, G. et al.
MK749913	RSVA/Homo sapiens/NIC/IIIn_16_0250_01NT/2016	2016	Nicaragua	NA1	Tan, G. et al.
MK749917	RSVA/Homo sapiens/NIC/IIIn_16_0265_01NT/2016	2016	Nicaragua	NA1	Tan, G. et al.
MN251607	GH100344/USA/2011	2011	USA	NA1	Chu, H. et al.
MN306017	SC0135	2018	USA	NA1	Lin, M.J. et al.
MN306021	SC0237	2018	USA	NA1	Lin, M.J. et al.
MN306029	SC0398	2019	USA	NA1	Lin, M.J. et al.
MN306030	SC0410	2019	USA	NA1	Lin, M.J. et al.
MN306031	SC0460	2019	USA	NA1	Lin, M.J. et al.
MN306045	SC0850	2019	USA	NA1	Lin, M.J. et al.
MN306048	SC0885	2019	USA	NA1	Lin, M.J. et al.
MN306050	SC1053	2019	USA	NA1	Lin, M.J. et al.
MN310477	SC0276	2018	USA	NA1	Lin, M.J. et al.
MN531557	GH300327/USA/2013	2013	USA	NA1	Chu, H. et al.
MN630090	RSVA/USA/UAMS-DID-009/2016	2016	USA	NA1	Kothari, A. et al.
MN630091	RSVA/USA/UAMS-DID-011/2016	2016	USA	NA1	Kothari, A. et al.
MN630092	RSVA/USA/UAMS-DID-015/2016	2016	USA	NA1	Kothari, A. et al.
MN630094	RSVA/USA/UAMS-DID-018/2016	2016	USA	NA1	Kothari, A. et al.
MN630099	RSVA/USA/UAMS-DID-028/2016	2016	USA	NA1	Kothari, A. et al.
MN630100	RSVA/USA/UAMS-DID-040/2016	2016	USA	NA1	Kothari, A. et al.
MN630101	RSVA/USA/ACRI-037/2016	2016	USA	NA1	Kothari, A. et al.
MN630102	RSVA/USA/ACRI-039/2016	2016	USA	NA1	Kothari, A. et al.
MN630103	RSVA/USA/ACRI-046/2016	2016	USA	NA1	Kothari, A. et al.
MN630105	RSVA/USA/ACRI-056/2016	2016	USA	NA1	Kothari, A. et al.
MN630106	RSVA/USA/ACRI-064/2016	2016	USA	NA1	Kothari, A. et al.

MN630107	RSVA/USA/ACRI-053/2016	2016	USA	NA1	Kothari, A. et al.
MT422269	RSVA/Novosibirsk/108Hp/2019	2019	Russia	NA1	Dubovitskiy, N.A. et al.
MT422270	RSVA/Novosibirsk/138Hp/2019	2019	Russia	NA1	Dubovitskiy, N.A. et al.
MT422271	RSVA/Novosibirsk/893Hp/2019	2019	Russia	NA1	Dubovitskiy, N.A. et al.
JX198112	Long	1956	USA		1
KJ155694	A2	1961	Australia		25
JX198143	CH-18537	1962	USA		Tapia, L.I. et al.
D00953	RB 94	1969	Belgium		26

References

1) Tapia, L.I.; Shaw, C.A.; Aideyan, L.O.; Jewell, A.M.; Dawson, B.C.; Haq, T.R.; Piedra, P.A. Gene sequence variability of the three surface proteins of human respiratory syncytial virus (HRSV) in Texas. *PLoS ONE* **2014**, *9*, E90786.
2) Rossey, I.; Gilman, M.S.; Kabeche, S.C.; Sedeyn, K.; Wrapp, D.; Kanekiyo, M.; Chen, M.; Mas, V.; Spitaels, J.; Melero, J.A.; Graham, B.S.; Schepens, B.; McLellan, J.S.; Saelens, X. Potent single-domain antibodies that arrest respiratory syncytial virus fusion protein in its prefusion state. *Nat. Commun.* **2017**, *8*, 14158.
3) Rebuffo-Scheer, C.; Bose, M.E.; He, J.; Khaja, S.; Ulatowski, M.; Beck, E.T.; Fan, J.; Kumar, S.; Nelson, M.I.; Henrickson, K.J. Whole genome sequencing and evolutionary analysis of human respiratory syncytial virus A and B from Milwaukee, WI 1998-2010. *PLoS ONE* **2011**, *6*, E25468.
4) Tan, L.; Lemey, P.; Houspie, L.; Viveen, M.C.; Jansen, N.J.; van Loon, A.M.; Wiertz, E.; van Bleek, G.M.; Martin, D.P.; Coenjaerts, F.E. Genetic variability among complete human respiratory syncytial virus subgroup A genomes: bridging molecular evolutionary dynamics and epidemiology. *PLoS ONE* **2012**, *7*, E51439.
5) Agoti, C.N.; Otieno, J.R.; Munywoki, P.K.; Mwihuri, A.G.; Cane, P.A.; Nokes, D.J.; Kellam, P.; Cotten, M. Local evolutionary patterns of human respiratory syncytial virus derived from whole-genome sequencing. *J. Virol.* **2015**, *89*, 3444-3454.
6) Widjojoatmodjo, M.N.; Boes, J.; van Bers, M.; van Remmerden, Y.; Roholl, P.J.; Luytjes, W. A highly attenuated recombinant human respiratory syncytial virus lacking the G protein induces long-lasting protection in cotton rats. *Virol. J.* **2010**, *7*, 114.
7) Xia, Q.; Zhou, L.; Peng, C.; Hao, R.; Ni, K.; Zang, N.; Ren, L.; Deng, Y.; Xie, X.; He, L.; Tian, D.; Wang, L.; Huang, A.; Zhao, Y.; Zhao, X.; Fu, Z.; Tu, W.; Liu, E. Detection of respiratory syncytial virus fusion protein variants between 2009 and 2012 in China. *Arch. Virol.* **2014**, *159*, 1089-1098.
8) Zhang, K.; He, J.; Li, C.; Bose, M.E.; Henrickson, K.J.; Zhou, J.; Zheng, B.J. Complete genome sequences of one human respiratory syncytial antigenic group a virus from china and its four mouse-adapted isolates. *Genome Announc.* **2015**, *3*, e00062-15.
9) Song, J.; Wang, H.; Ng, T.I.; Cui, A.; Zhu, S.; Huang, Y.; Sun, L.; Yang, Z.; Yu, D.; Yu, P.; Zhang, H.; Zhang, Y.; Xu, W. Sequence analysis of the fusion protein gene of human respiratory syncytial virus circulating in China from 2003 to 2014. *Sci. Rep.* **2018**, *8*, 17618.
10) Grad, Y.H.; Newman, R.; Zody, M.; Yang, X.; Murphy, R.; Qu, J.; Malboeuf, C.M.; Levin, J.Z.; Lipsitch, M.; DeVincenzo, J. Within-host whole-genome deep sequencing and diversity analysis of human respiratory syncytial virus infection reveals dynamics of genomic diversity in the absence and presence of immune pressure. *J. Virol.* **2014**, *88*, 7286-7293.
11) Do,L. A.; Wilm, A.; van Doorn, H.R.; Lam, H.M.; Sim, S.; Sukumaran, R.; Tran, A.T.; Nguyen, B.H.; Tran, T.T.; Tran, Q.H.; Vo, Q.B.; Tran Dac, N.A.; Trinh, H.N.; Nguyen, T.T.; Le Binh, B.T.; Le, K.; Nguyen, M.T.; Thai, Q.T.; Vo, T.V.; Ngo, N.Q.; Dang, T.K.; Cao, N.H.; Tran, T.V.; Ho, L.V.; Farrar, J.; De Jong, M.; Chen, S.; Nagarajan, N.; Bryant, J.E.; Hibberd, M.L. Direct whole-genome deep-sequencing of human respiratory syncytial virus A and B from Vietnamese children identifies distinct patterns of inter- and intra-host evolution. *J. Gen. Virol.* **2015**, *96*, 3470-3483.
12) Chen, X.; Xu, B.; Guo, J.; Li, C.; An, S.; Zhou, Y.; Chen, A.; Deng, L.; Fu, Z.; Zhu, Y.; Liu, C.; Xu, L.; Wang, W.; Shen, K.; Xie, Z. Genetic variations in the fusion protein of respiratory syncytial virus isolated from children hospitalized with community-acquired pneumonia in China. *Sci. Rep.* **2018**, *8*, 4491.
13) Di Giallonardo, F.; Kok, J.; Fernandez, M.; Carter, I.; Geoghegan, J.L.; Dwyer, D.E.; Holmes, E.C.; Eden, J.S. Evolution of human respiratory syncytial virus (RSV) over multiple seasons in New South Wales, Australia. *Viruses* **2018**, *10*, E476.
14) Lee, W.J.; Kim, Y.J.; Kim, D.W.; Lee, H.S.; Lee, H.Y.; Kim, K. Complete genome sequence of human respiratory syncytial virus genotype A with a 72-nucleotide duplication in the attachment protein G gene. *J. Virol.* **2012**, *86*, 13810-13811.
15) Choudhary, M.L.; Wadhwa, B.S.; Jadhav, S.M.; Chadha, M.S. Complete genome sequences of two human respiratory syncytial virus genotype A strains from India, RSV-A/NIV1114046/11 and RSV-A/NIV1114073/11. *Genome Announc.* **2013**, *1*, e00165-13.
16) Gu, H.; Xie, Z.; Li, T.; Zhang, S.; Lai, C.; Zhu, P.; Wang, K.; Han, L.; Duan, Y.; Zhao, Z.; Yang, X.; Xing, L.; Zhang, P.; Wang, Z.; Li, R.; Yu, J.J.; Wang, X.; Yang, P. Angiotensin-converting enzyme 2 inhibits lung injury induced by respiratory syncytial virus. *Sci. Rep.* **2016**, *6*, 19840.
17) Ren, L.; Xia, Q.; Xiao, Q.; Zhou, L.; Zang, N.; Long, X.; Xie, X.; Deng, Y.; Wang, L.; Fu, Z.; Tian, D.; Zhao, Y.; Zhao, X.; Li, T.; Huang, A.; Liu, E. The genetic variability of glycoproteins among respiratory syncytial virus subtype A in China between 2009 and 2013. *Infect. Genet. Evol.* **2014**, *27*, 339-347.
18) Yun, M.R.; Kim, A.R.; Lee, H.S.; Kim, D.W.; Lee, W.J.; Kim, K.; Kim, S.S.; Kim, Y.J. Complete genome sequences of human respiratory syncytial virus genotype A and B isolates from South Korea. *Genome Announc.* **2015**, *3*, e00332-15.
19) Fu, X.; Cheng, Y.; He, Z.; Dong, W.; Lan, K.; Zhang, C.; Hu, Y. Complete genome sequence of human respiratory syncytial virus from Shanghai, China. *Genome Announc.* **2015**, *3*, e00989-15.
20) Gong, Y.N.; Yang, S.L.; Chen, G.W.; Chen, Y.W.; Huang, Y.C.; Ning, H.C.; Tsao, K.C. A metagenomics study for the identification of respiratory viruses in mixed clinical specimens: an application of the iterative mapping approach. *Arch. Virol.* **2017**, *162*, 2003-2012.
21) Malasao, R.; Furuse, Y.; Okamoto, M.; Dapat, C.; Saito, M.; Saito-Obata, M.; Tamaki, R.; Segubre-Mercado, E.; Lupisan, S.; Oshitani, H. Complete genome sequences of 13 human respiratory syncytial virus subgroup A strains of genotypes NA1 and ON1 isolated in the Philippines. *Genome Announc.* **2018**, *6*, e00151-18.
22) Okamoto, M.; Dapat, C.P.; Sandagon, A.M.D.; Batangan-Nacion, L.P.; Lirio, I.C.; Tamaki, R.; Saito, M.; Saito-Obata, M.; Lupisan, S.P.; Oshitani, H. Molecular characterization of respiratory syncytial virus in children with repeated infections with subgroup B in the Philippines. *J. Infect. Dis.* **2018**, *218*, 1045-1053.
23) Goya, S.; Valinotto, L.E.; Tittarelli, E.; Rojo, G.L.; Nabaes Jodar, M.S.; Greninger, A.L.; Zaiat, J.J.; Marti, M.A.; Mistchenko, A.S.; Viegas, M. An optimized methodology for whole genome sequencing of RNA respiratory viruses from nasopharyngeal aspirates. *PLoS ONE* **2018**, *13*, e0199714.
24) Otieno, J.R.; Kamau, E.M.; Oketch, J.W.; Ngoi, J.M.; Gichuki, A.M.; Binter, S.; Otieno, G.P.; Ngama, M.; Agoti, C.N.; Cane, P.A.; Kellam, P.; Cotten, M.; Lemey, P.; Nokes, D.J. Whole genome analysis of local Kenyan and global sequences unravels the epidemiological and molecular evolutionary dynamics of RSV genotype ON1 strains. *Virus Evol.* **2018**, *4*, vey027.
25) Patton, K.; Aslam, S.; Lin, J.; Yu, L.; Lambert, S.; Dawes, G.; Esser, M.T.; Woo, J.; Janetzki, S.; Cherukuri, A. Enzyme-linked immunospot assay for detection of human respiratory syncytial virus f protein-specific gamma interferon-producing T cells. *Clin. Vaccine Immunol.* **2014**, *21*, 628-635.
26) Baybutt, H.N.; Pringle, C.R. Molecular cloning and sequencing of the F and 22K membrane protein genes of the RSS-2 strain of respiratory syncytial virus. *J. Gen. Virol.* **1987**, *68 (Pt 11)*, 2789-2796.

Supplementary Table S2. Evolutionary rates for each genotype. As noted, the genotype GA6 was not examined due to small strain numbers (6 strains).

	Evolutionary rates (95%HPD) (substitutions/site/year)
All RSV-A (1,465 strains)	7.69×10^{-4} (7.10×10^{-4} - 8.29×10^{-4})
GA1 (35 strains)	4.48×10^{-4} (2.79×10^{-4} - 6.24×10^{-4})
GA2 (77 strains)	8.34×10^{-4} (6.43×10^{-4} - 1.04×10^{-3})
GA3 (28 strains)	8.02×10^{-4} (5.15×10^{-4} - 1.09×10^{-3})
GA4 (17 strains)	6.04×10^{-4} (2.22×10^{-4} - 1.00×10^{-3})
GA5 (174 strains)	7.11×10^{-4} (5.82×10^{-4} - 8.34×10^{-4})
GA7 (34 strains)	7.56×10^{-4} (4.97×10^{-4} - 1.02×10^{-3})
NA1 (1,092 strains)	7.61×10^{-4} (6.85×10^{-4} - 8.38×10^{-4})

Supplementary Table S3. Phylogenetic distances for each genotype.

	Phylogenetic distance (mean±SD)
All RSV-A (1,465 strains)	0.024±0.021
GA1 (35 strains)	0.006±0.004
GA2 (77 strains)	0.012±0.006
GA3 (28 strains)	0.017±0.007
GA4 (17 strains)	0.010±0.004
GA5 (174 strains)	0.010±0.004
GA6 (6 strains)	0.007±0.004
GA7 (34 strains)	0.011±0.006
NA1 (1,092 strains)	0.008±0.003
